# Anti-orthopoxvirus drugs inhibit lumpy skin disease virus replication by targeting viral DNA polymerase

**DOI:** 10.1371/journal.ppat.1013903

**Published:** 2026-01-26

**Authors:** Zuxin Gong, Jiaqi Dai, Hailong Qu, Yongxin Hu, Fanqi Sun, Chenchen Liu, Xin Li, Chunyan Feng, Zhiliang Wang, Zhen Yang, Gongguan Liu

**Affiliations:** 1 Key Laboratory of Animal Diseases Diagnostic and Immunology, Ministry of Agriculture, MOE International Joint Collaborative Research Laboratory for Animal Health & Food Safety, The Belt and Road International Sci-Tech Innovation Institute of Transboundary Animal Disease Diagnosis and Immunization, College of Veterinary Medicine, Nanjing Agricultural University, Nanjing, China; 2 China Animal Health and Epidemiology Center, Qingdao, China; 3 Chinese Academy of Quality and Inspection & Testing, Beijing, China; Cardiff University, UNITED KINGDOM OF GREAT BRITAIN AND NORTHERN IRELAND

## Abstract

Lumpy skin disease (LSD) is an emerging transboundary disease caused by lumpy skin disease virus (LSDV), posing significant threats to global cattle health in the absence of validated drugs. Here, we constructed a recombinant LSDV (rLSDV) expressing both mCherry and luciferase reporters for high-throughput drug screening, and the rLSDV retained virological characteristics phenotypically indistinguishable from the parental strain, with the reporter genes serving as precise and sensitive quantitative indicators for viral replication. Leveraging this platform, we identified six candidates from a library of anti-orthopoxvirus compounds, namely cytarabine (AraC), enrofloxacin (ENR), idoxuridine (IDU), fialuridine (FIAU), ribavirin (RBV), and vidarabine (AraA), demonstrating high antiviral activity concomitant with low cytotoxicity. Time-of-addition experiments revealed that all identified candidates primarily inhibited the viral replication phase. Mechanistical analysis revealed that anti-LSDV agents suppressed synthesis of both viral and host DNA and/or RNA. In particular, AraC markedly blocked viral DNA synthesis and prevented activation of viral late gene promoters, thereby arresting the replication cycle at an early stage. Structural alignment data suggested that AraC may bind to the viral DNA polymerase at residues D554, R639, K666, N670, and D758 to inhibit its activity. Notably, AraC induced only minimal host DNA damage and apoptosis, and host DNA synthesis gradually recovered during treatment, although these residues are conserved in bovine DNA polymerase. Hence, the mechanistic landscape delineated herein, together with the established clinical availability of the anti-orthopoxvirus agents, underscore their potential as repurposable therapeutics for LSDV infection.

## Author summary

Lumpy skin disease virus (LSDV) threatens cattle production worldwide, yet limited knowledge of its pathogenic mechanisms has hindered the development of effective antiviral therapies. To address the lack of approved drugs, we established a sensitive real-time high-throughput screening platform and identified six nucleotide analogs from an anti-orthopoxvirus compound library that interfere with viral nucleotide synthesis, highlighting viral DNA or RNA polymerase as viable antiviral targets. Among them, AraC showed exclusively high antiviral activity with a wider safe concentration range. Mechanistically, AraC is predicted to target the viral DNA polymerase, leading to suppressed viral DNA synthesis and the consequent completely blockade of viral late protein expression. Given their existing clinical use in human and veterinary medicine, these compounds represent promising lead candidates for developing effective therapies against LSD.

## Introduction

Lumpy skin disease (LSD) is a subacute to acute infectious disease caused by the lumpy skin disease virus (LSDV), which belongs to the *Capripoxvirus* (CaPV) genus within the *Poxviridae* family [1]. The genome of LSDV is a double-stranded DNA approximately 145–152 kb in length, encoding about 156 open reading frames (ORFs). These ORFs include the DNA polymerase required for viral replication and the RNA polymerase essential for transcriptional processes. LSDV exhibits high contagiousness among all cattle breeds and is primarily transmitted through arthropods, with potential for long-distance wind-assisted spread [2,3]. Epidemiological data indicates an approximate 26% morbidity rate and 7.5% mortality rate in affected herds [4]. Infected cattle typically present with fever, generalized cutaneous nodular lesions, often accompanied by weight loss, reduced milk production, and reproductive dysfunction, leading to significant economic losses [4,5]. Due to its substantial impact, the World Organization for Animal Health (WOAH) has classified LSD as a notifiable animal disease. Since 2019, recombinant strains have emerged in Asian regions [6–9], and within just one year, the epidemic caused by recombinant strains had already inflicted economic losses totaling up to $1.46 billion in Asia [10]. Currently, the LSD epidemic continues to spread, posing a global challenge for disease control and prevention.

To date, there are no effective prophylactic or therapeutic drugs available for LSD [11]. The primary control strategy relies on a combination of emergency vaccination with live-attenuated vaccines and culling of infected herds. However, large-scale culling will lead to significant economic losses, and live-attenuated vaccination may induce adverse reactions in cattle and may increase the risk of viral recombination. Therefore, although vaccination remains critical for outbreak containment, the development of highly effective small-molecule antiviral drugs is an essential complementary strategy to reduce LSDV-associated mortality, morbidity, as well as economic losses.

In drug development for emerging infectious diseases, the strategy of drug repurposing has demonstrated unique advantages. Recent outbreaks of SARS-CoV-2 and monkeypox virus (MPXV) have posed severe threats to global public health [12,13], yet traditional drug development often requires decades, making it difficult to respond promptly to such emerging challenges. Notably, several repurposed antiviral agents have demonstrated efficacy against SARS-CoV-2 or MPXV infections in recent studies [14–17], suggesting that screening existing antiviral compound libraries may serve as a viable strategy for rapidly developing therapeutics for LSD. This approach would not only substantially shorten the development timeline but also significantly reduce associated costs.

LSDV encodes a large number of viral proteins, yet the functions of only a few have been elucidated to date [18–20]. This limited functional characterization not only hinders a deeper understanding of its pathogenic mechanisms but also restricts the identification of antiviral drug targets. As a member of the *Poxviridae* family, LSDV relies on its own DNA polymerase to replicate the viral genome, making polymerase-associated replication steps the critical points for therapeutic intervention. The Orthopoxvirus genus is the most extensively studied within the *Poxviridae* family, and numerous compounds with anti-orthopoxvirus activity have been identified [21,22]. Comparative genomic analyses have revealed that LSDV shares approximately 49% average amino acid sequence identity with vaccinia virus (VACV), a prototypical orthopoxvirus, and the sequence identity of their DNA polymerases is even higher at 66% [1], suggesting evolutionary conservation in essential functional proteins and replication mechanisms. Based on these foundations, we established a high-throughput screening platform specifically tailored for LSDV drug discovery and employed a drug repurposing strategy to rapidly and cost-effectively identify potential therapeutic candidates from an anti-orthopoxvirus compound library. This approach provides a foundation for the development of antiviral therapeutics against LSDV.

## Results

### Establishment of a high-throughput screening platform for LSDV inhibitors

**Fig 1:**
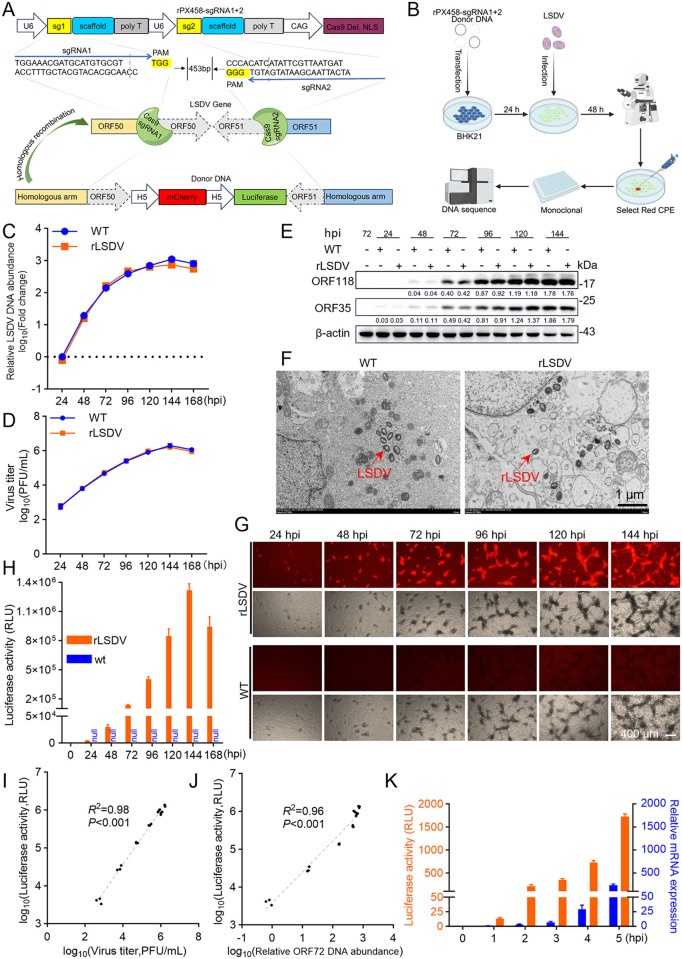
Construction and characterization of the recombinant LSDV reporter virus. (A) The schematic illustration of the core elements of rPX458-sgRNA1 + 2 and the donor plasmid. (B) The schematic illustration of the construction and purification of rLSDV. This figure was created with BioRender.com. (C-H) MDBK cells were infected with either WT LSDV or rLSDV at a multiplicity of infection (MOI) of 0.01; viral titers (C), relative viral DNA abundance (D), and the expression of the viral early protein ORF35 and late protein ORF118 (E) were measured at the indicated time points; the morphology of viruses were compared at 72 hpi by TEM; the red arrow points to a typical virus particle (F); mCherry fluorescence was monitored from 24 to 144 hpi (G), and RLU was measured from 24 to 168 hpi (H). (I and J) Pearson correlation analysis was performed to assess the relationship between luciferase activity and viral titer (I), as well as between luciferase activity and relative DNA abundance (J). *R*² indicates the goodness of fit, and *P* values denote the significance of the correlation. (K) MDBK cells were infected with rLSDV at an MOI of 0.1, and the sensitivity of the two detection methods was compared during 0-5 hpi; viral *ORF72* mRNA expression levels relative to bovine *β-actin* mRNA were quantified by qPCR, while luciferase activity was measured. The grayscale intensity of key protein bands in (E) was normalized to the intensity of the internal control. Scale bars are shown in the lower right corner in (F) and (G). Data are presented as mean ± SD, n = 3.

To develop a high-throughput screening system for anti-LSDV drug discovery, we engineered a recombinant LSDV (rLSDV) strain by inserting the mCherry and firefly luciferase reporter genes into the viral genome (Fig 1A and 1B), enabling real-time imaging and quantitative analysis of viral replication. To evaluate whether the insertion of fluorescent reporter gene affects the replication characteristics and virion assembly of LSDV, we compared the growth kinetics of the wild-type LSDV (WT LSDV) and the rLSDV using plaque assays, qPCR, and western blot. The results demonstrated no significant differences in replication dynamics between rLSDV and WT LSDV (Fig 1C, 1D and 1E). The virion morphological features of the two viruses remained consistent, as illustrated by electron microscopy (Fig 1F). These findings suggest that rLSDV retains the biological characteristics of WT LSDV.

To validate the specificity of reporter genes in imaging detection, we analyzed the colocalization of mCherry fluorescence signals with virus-induced cytopathic effects (CPE) under fluorescence microscopy. The results showed that as the infection progressed, the mCherry fluorescence area expanded synchronously and completely overlapped with the CPE region (Fig 1G). Meanwhile, the firefly luciferase activity measured in relative light unit (RLU) increased over time in the rLSDV-infected cells (Fig 1H). No mCherry fluorescence signal or luciferase activity was detected in the WT LSDV-infected group at consecutive time points. To further evaluate the correlation between luciferase activity and viral replication levels, we performed correlation analyses on luciferase activity, viral titer, and relative viral DNA abundance in samples collected at different time points post rLSDV infection. The results demonstrated a significant positive correlation between luciferase activity and viral titer (*R*^2^ = 0.98, *P* < 0.001) (Fig 1I) or relative viral DNA abundance (*R*^2^ = 0.96, *P* < 0.001) (Fig 1J). These findings confirm that mCherry and luciferase expressed by rLSDV accurately reflect the replication levels of LSDV.

To assess the sensitivity of rLSDV detection, we measured luciferase activity produced by rLSDV within 5 hours post infection (hpi) while simultaneously detecting the expression of the viral early gene *ORF72* via qPCR. The results revealed that both luciferase activity and viral *ORF72* expression increased synchronously as early as 1 hpi, of which the luciferase assay exhibits higher sensitivity comparable to qPCR (Fig 1K).

### Identification of anti-LSDV compounds from anti-orthopoxvirus drug library

To expedite anti-LSDV drug discovery, we conducted a targeted screening of anti-orthopoxvirus drug library, considering the substantial genomic homology and phylogenetic conservation between LSDV and VACV [1]. Additionally, we also included ivermectin in our screening panel as an internal control, since it has been shown to inhibit LSDV replication in Vero cells [23].

**Fig 2:**
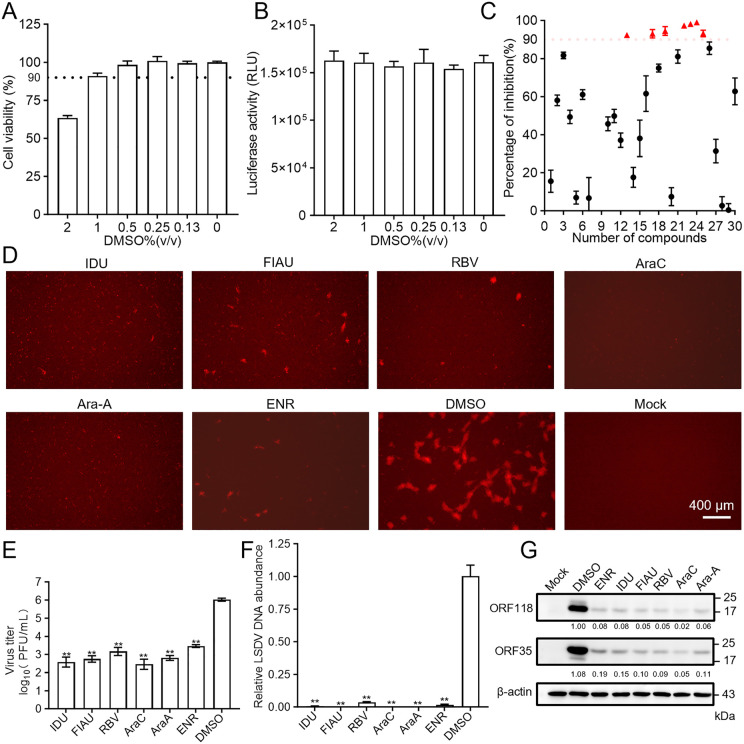
Screening of anti-LSDV compounds from an anti-orthopoxvirus drug library identifies six replication inhibitors. (A) MDBK cells were treated with different concentrations of DMSO for 72 h, and cell viability was determined by CCK-8 assay. (B) rLSDV-infected (0.1 MOI) MDBK cells were treated with different concentrations of DMSO for 72 h, followed by measurement of luciferase activity. (C and D) rLSDV-infected (0.1 MOI) MDBK cells were treated with candidate compounds at concentrations indicated in Table 1 for 72 h, and antiviral activity was evaluated by luciferase activity (C) and mCherry fluorescence intensity (D). (E to G) MDBK cells were infected with WT LSDV (0.1 MOI), and were simultaneously treated with ENR (100 μM), IDU (20 μM), FIAU (5 μM), RBV (20 μM), AraC (0.5 μM), AraA (20 μM) or DMSO for 72 h. Antiviral efficacy was confirmed again through plaque assay for viral titers (E), qPCR for relative viral DNA abundance normalized to host *β-actin* (F), and western blot (G) analysis of viral protein expression (early protein ORF35 and late protein ORF118). Scale bars are shown in the lower right corner in (D). Data are presented as mean ± SD, n = 3. Two-sided Student’s t-test was used for statistical analysis in (E) and (F); **P < 0.01. The grayscale intensity of key protein bands in (G) was normalized to the intensity of the internal control. Data are presented as mean ± SD, n = 3.

Given that the solvent for compounds in the library is dimethyl sulfoxide (DMSO), we first analyzed the cytotoxic effects of DMSO on MDBK and its impact on viral replication. The results showed that ≤ 1% DMSO in the culture medium maintained > 90% cell viability without significantly affecting viral replication (Fig 2A and 2B). To avoid confounding effects of DMSO toxicity and considering that the stock concentration of compounds was 10 mM, the maximum test concentration was kept at 100 μM (1% final DMSO). Since the effective concentrations of different compounds may vary significantly, we first tested the half-maximum cytotoxic concentration (CC_50_) of all compounds. The concentrations preserving > 50% cell viability were designated as screening concentrations for primary screening (Table 1).

**Table 1:** Primary screening results. The stored concentration of Amantadine was 2 mM. The inhibition rate represents expressed as mean ± SD, n = 3. For a single experiment, the inhibition rate is calculated as (1 - (luciferase activity of the compound group/ luciferase activity of the DMSO group)) × 100%.

Drug No.	Drug Name	Initial screening concentrations (μM)	Inhibition (%)	CAS No.
1	Thymidine	100	15.53 ± 5.81	50-89-5
2	Amantadine (hydrochloride)	100	58.03 ± 2.25	665-66-7
3	Adefovir dipivoxil	0.5	81.69 ± 1.39	142340-99-6
4	Levofloxacin	100	49.37 ± 2.91	100986-85-4
5	Hydroxyurea	100	7.01 ± 2.82	127-07-1
6	Clevudine	100	61.09 ± 2.12	163252-36-6
7	Ofloxacin	100	6.74 ± 8.78	82419-36-1
8	Thiosemicarbazide	100	-1.53 ± 4.03	79-19-6
9	Novobiocin (sodium)	100	-0.38 ± 2.33	1476-53-5
10	3’-Fluoro-3’-deoxythymidine	100	45.74 ± 3.01	25526-93-6
11	5’-Deoxyadenosine	100	49.81 ± 2.91	4754-39-6
12	Carbenoxolone (disodium)	100	37.12 ± 3.05	7421-40-1
13	Enrofloxacin	100	92.32 ± 1.07	93106-60-6
14	2-Aminoquinoline	100	17.66 ± 4.25	580-22-3
15	Mitoxantrone	0.01	38.03 ± 7.84	65271-80-9
16	Mitoxantrone (dihydrochloride)	0.01	61.53 ± 7.64	70476-82-3
17	Idoxuridine	100	93.01 ± 1.78	54-42-2
18	Trifluridine	0.25	74.99 ± 1.73	70-00-8
19	Fialuridine	100	94.46 ± 1.96	69123-98-4
20	Levofloxacin (hydrate)	100	7.41 ± 3.85	138199-71-0
21	Rifampicin	25	81.06 ± 2.90	13292-46-1
22	Ribavirin	100	97.24 ± 0.32	36791-04-5
23	Cytarabine	0.5	98.08 ± 0.92	147-94-4
24	Vidarabine	100	99.02 ± 0.12	5536-17-4
25	Enrofloxacin (monohydrochloride)	100	93.18 ± 1.44	93106-59-3
26	Netropsin (dihydrochloride)	100	85.47 ± 2.69	18133-22-7
27	Cidofovir	100	31.37 ± 5.07	113852-37-2
28	Phosphonoacetic acid	100	2.76 ± 3.78	4408-78-0
29	Amantadine	20	0.47 ± 2.73	768-94-5
30	Ivermectin	3	62.82 ± 5.78	70288-86-7

The preliminary screening results revealed that six candidate compounds - enrofloxacin (ENR), idoxuridine (IDU), fialuridine (FIAU), ribavirin (RBV), cytarabine (AraC), and vidarabine (AraA) - exhibited potent inhibitory effects against LSDV replication, with inhibition rates exceeding 90% as determined by luciferase activity (Fig 2C). Consistent results were observed in mCherry fluorescence imaging (Fig 2D). To further validate the anti-LSDV effects of these candidates, we repurchased the compounds from an alternative supplier (TargetMol, USA), and tested their effects against WT LSDV. As shown in Fig 2E, plaque assays in MDBK cells revealed that the mean viral titers in the DMSO-treated group were 2,462-fold, 1,778-fold, 667-fold, 3,200-fold, 1,600-fold, and 360-fold higher than those in the IDU-, FIAU-, RBV-, AraC-, AraA-, and ENR-treated groups, respectively. Consistently, qPCR (Fig 2F) and western blot (Fig 2G) results further confirmed the potent antiviral activities of these compounds.

### Assessment of the therapeutic potential using the selectivity index

**Fig 3:**
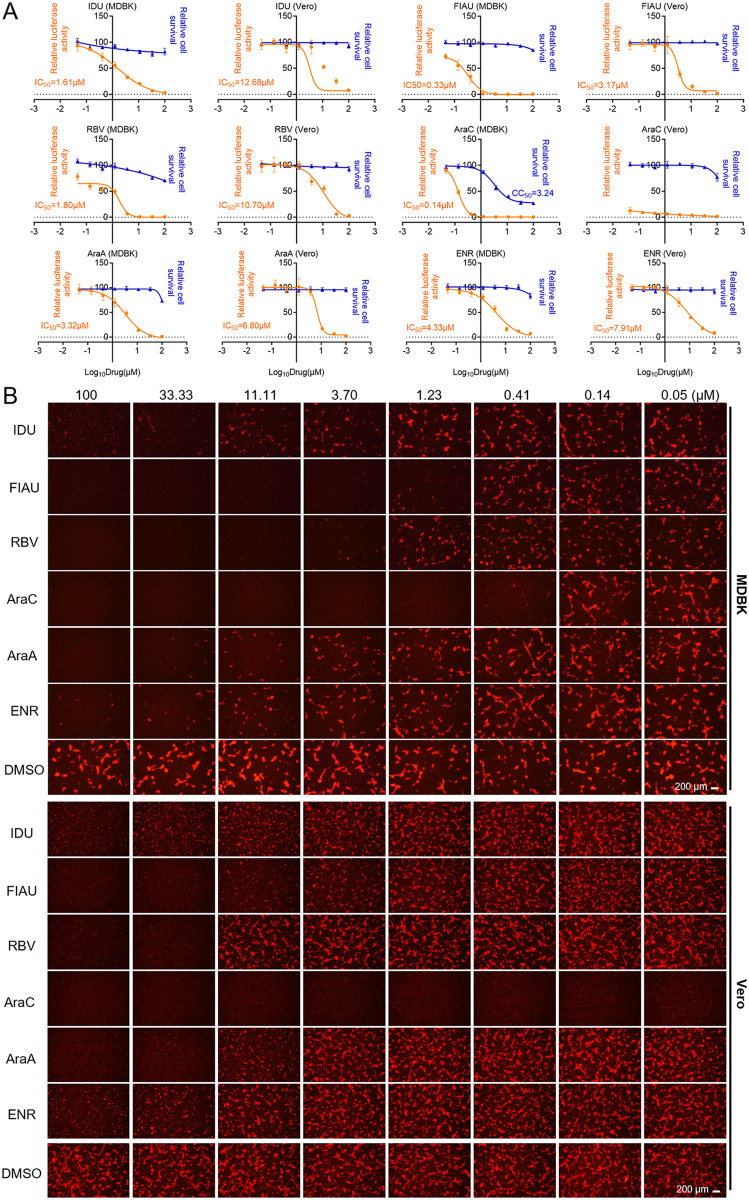
CC_50_ and IC_50_ values of candidate compounds in MDBK and Vero cells. (A) Cells were treated with 3-fold serially diluted compounds for 72 h, cell viability was determined by CCK-8 assay, dose-inhibition curves were generated using GraphPad Prism 7 to calculate CC_50_. rLSDV-infected (0.1 MOI) cells were treated with three-fold serially diluted compounds for 72 h, and viral replication was assessed by luciferase activity; dose-inhibition curves were generated using GraphPad Prism 7 to calculate IC_50_. (B) mCherry fluorescence signals were concurrently recorded for validation. Scale bars are shown in the lower right corner in (B). Data are presented as mean ± SD, n = 3.

To further assess the antiviral potential of these six compounds, we calculated the half-maximal inhibitory concentration (IC_50_) by measuring the luciferase activity in rLSDV-infected MDBK cells (Figs 3A and S1). Parallel monitoring of mCherry fluorescence expression by fluorescence microscopy demonstrated a concentration-dependent decrease in viral replication (Fig 3B). The same experiment was applied to Vero cells to determine CC_50_ and IC_50_ values (Fig 3A) and to examine mCherry fluorescence patterns, which also revealed a concentration-dependent decrease in viral replication (Fig 3B). To evaluate the therapeutic potential of these compounds against LSDV, we calculated the selectivity index (SI, SI = CC_50_/ IC_50_) in both cell lines. All tested compounds had SI values greater than 5 (Table 2), demonstrating high safety profiles at effective concentrations.

**Table 2:** Selectivity index of candidate compounds.

	MDBK			Vero		
	CC_50_ (μM)	IC_50_ (μM)	SI	CC_50_ (μM)	IC_50_ (μM)	SI
FIAU	>100	>0.33	>303.03	>100	3.17	>31.54
IDU	>100	>1.61	>62.11	>100	12.68	>7.89
RBV	>100	>1.8	>55.56	>100	10.7	>9.35
AraA	>100	>3.32	>30.12	>100	6.8	>14.71
AraC	3.24	0.14	23.14	>100	<0.05	>2000
ENR	>100	>4.33	>23.09	>100	7.91	>12.64

### The candidate compounds inhibit LSDV infection during the viral replication phase

**Fig 4:**
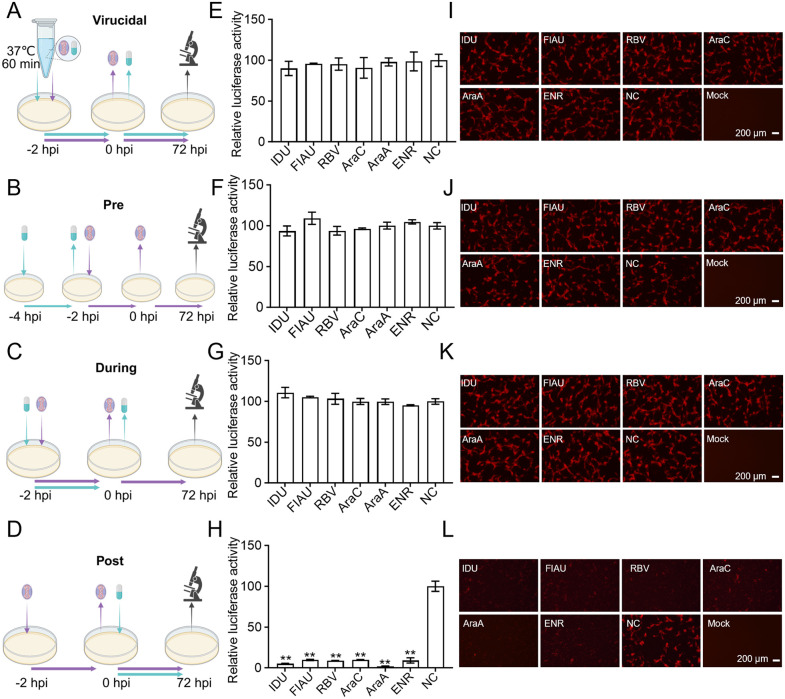
Time-of-addition assay for anti-LSDV activity of hit compounds in MDBK cells. (A-D) Schematic illustration of the time-of-addition experiment. These figures were created with BioRender.com. MDBK cells were infected with rLSDV (0.1 MOI) and treated with ENR (100 μM), IDU (20 μM), FIAU (5 μM), RBV (20 μM), AraC (0.5 μM), or AraA (20 μM) at four distinct phases (Virucidal, Pre, During and Post); (E-H) Antiviral effects were quantified by relative luciferase activity; (I-L) mCherry fluorescence were simultaneously captured. Scale bars are shown in the lower right corner in (I, J, K and L). Data are presented as mean ± SD, n = 3. **P < 0.01.

To elucidate which stage of viral infection is targeted by these six candidate compounds, we performed time-of-addition experiments. Viruses were pre-incubated with compounds for 1 h to examine direct virucidal effect (Fig 4A). In parallel, compounds were added into MDBK cell culture at pre-infection (Pre) to assess receptor blockade (Fig 4B), at during-infection (During) to evaluate viral entry inhibition (Fig 4C), and at post-infection (Post) to analyze suppression of viral replication (Fig 4D). Notably, none of the compounds significantly inhibited luciferase activity when added pre-incubation with virus (Fig 4E), pre-infection (Fig 4F), or during infection (Fig 4G). In contrast, substantial inhibition of luciferase activity was observed when compounds were administered post-infection (Fig 4H). These results were corroborated by the mCherry fluorescence signal (Fig 4I–L). Collectively, these results demonstrate that ENR, IDU, FIAU, RBV, AraC, and AraA primarily inhibit LSDV replication rather than receptor blockade, viral attachment or entry, and virucidal effect.

### Anti-LSDV candidates suppress viral DNA synthesis

**Fig 5:**
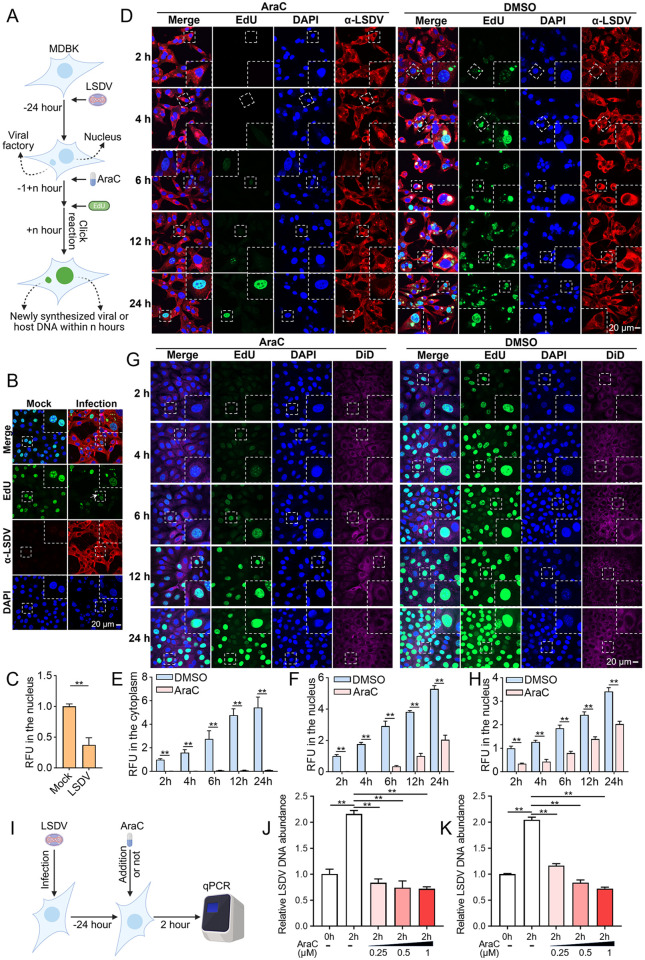
AraC suppresses LSDV DNA synthesis. (A) Schematic diagram of the EdU-labeling assay used to detect newly synthesized viral and host DNA during LSDV infection. This figure was created with BioRender.com. (B and C) MDBK cells were infected with LSDV at 1 MOI. At 24 hpi, cells were incubated with AraC (0.5 μM) for 1 h, followed by an additional 1 h incubation with AraC in the continued presence of EdU to label newly synthesized DNA. Host DNA synthesis was visualized by fluorescence microscopy (B), in which white arrows indicate representative foci of newly synthesized viral DNA. Nuclear EdU relative fluorescence unit (RFU) values were quantified using ImageJ (C), in which viral proteins were detected using rabbit anti-LSDV polyclonal antibodies. (D-F) MDBK cells were infected with LSDV at 1 MOI. At 24 hpi, cells were treated with AraC (0.5 μM) for 1 h, followed by incubation with EdU for the indicated times in the continued presence of AraC (0.5 μM). Viral and host DNA synthesis was assessed by fluorescence microscopy (D). Cytoplasmic (E) and nuclear (F) EdU RFU values were quantified using ImageJ. Viral proteins were detected using rabbit anti-LSDV polyclonal antibodies. (G and H) MDBK cells were treated with AraC (0.5 μM) or an equivalent volume of DMSO for 1 h, followed by incubation with EdU for the indicated times in the continued presence of AraC (0.5 μM) to label newly synthesized DNA. Host DNA synthesis was examined by fluorescence microscopy (G), and nuclear EdU RFU values were quantified using ImageJ (H). (I) Schematic diagram of the qPCR assay used to analyze viral DNA replication during LSDV infection and under AraC treatment. This figure was created with BioRender.com. (J and K) MDBK and Vero cells were infected with LSDV at an MOI of 1. At 24 hpi, cells were treated with the indicated concentrations of AraC for 2 h. Viral replication in MDBK (J) and Vero (K) cells was quantified by qPCR and analyzed using the 2^–ΔΔCt method after normalization to host *β-actin*. Scale bars are shown in the lower right corner in (B, D and G). Data are presented as mean ± SD, n = 3. **P < 0.01.

LSDV employs its encoded DNA polymerase to mediate viral DNA synthesis. To evaluate the effects of five nucleoside analogs (IDU, FIAU, RBV, AraC, and AraA) on viral DNA synthesis, we followed the workflow depicted in Fig 5A and used an EdU labeling method to simultaneously detect newly synthesized viral and host DNA in both virus and host cells. As a thymidine analog that can be fluorescently labeled via click reaction, the signal intensity of EdU is positively correlated with the amount of DNA synthesis [24]. Given the cytoplasmic replication of LSDV, newly synthesized viral DNA was indicated by EdU signals localized in the cytoplasm, whereas host DNA synthesis was reflected by EdU signals predominantly in the nucleus (with negligible contribution from mitochondrial DNA).

Confocal microscopy analysis revealed that cytoplasmic EdU signals were observed exclusively in LSDV-infected cells (Fig 5B), confirming the validity of the experimental approach. Compared to the mock group, LSDV infection significantly reduced nuclear EdU signal intensity (Fig 5B and 5C), suggesting that LSDV infection inhibits host DNA synthesis. During viral infection, the addition of AraC suppressed EdU signals in both the cytoplasm (Fig 5D and 5E) and the nucleus (Fig 5D and 5F). Notably, with prolonged AraC exposure, the nuclear EdU signal gradually intensified, whereas the cytoplasmic signal remained consistently low. In uninfected cells, treatment with AraC markedly reduced the nuclear EdU signal compared with the control group (Fig 5G and 5H). However, as the treatment duration increased, a noticeable reappearance of nuclear EdU signals was observed (Fig 5G and 5H). These observations indicate that AraC inhibits DNA synthesis of both the host and the virus, but exerts a more sustained and stable inhibitory effect on LSDV DNA replication. Using the same approach, we evaluated the effects of other compounds. IDU, FIAU, and AraA all exhibited significant inhibitory effects on both viral and host DNA synthesis, whereas ENR and RBV showed no apparent inhibitory activity (S2A–2C Fig).

To further validate the inhibitory effect of AraC on LSDV DNA synthesis, we quantified relative viral DNA abundance by qPCR following the scheme shown in Fig 5I. After 2 h of AraC treatment, LSDV DNA levels showed no appreciable increase in either MDBK (Fig 5J) or Vero cells (Fig 5K), whereas the DMSO controls exhibited a marked rise (Fig 5J and 5K). These data substantiate that AraC effectively suppresses LSDV DNA synthesis.

### Anti-LSDV candidates suppress viral RNA synthesis, with the exception of AraC, at effective antiviral levels

To assess the impact of ENR, IDU, FIAU, RBV, AraC, and AraA on viral RNA synthesis, we employed EU labeling to detect newly synthesized RNA of viral and host cells. EU, a uridine analog, exhibits a signal intensity that correlates positively with RNA synthesis levels via click reaction [25]. Given that LSDV replicates in the cytoplasm, EU signals corresponding to newly synthesized viral RNA were predominantly localized in the cytoplasm. Although eukaryotic RNA transcription primarily occurs in the nucleus, newly synthesized host RNA is subsequently transported to the cytoplasm for functional purposes [26], which may interfere with viral RNA detection. To minimize this interference, the detection was performed after 1 h EU treatment, as cytoplasmic levels of newly synthesized host RNA remain low at this early time point (S3 Fig), thereby minimizing its impact on viral RNA detection.

Confocal microscopy analysis revealed few cytoplasmic EU signals in mock-treated cells (S4A Fig). Thus, at the same time point, the cytoplasmic EU signals in LSDV-infected cells primarily reflected newly synthesized viral RNA. No significant difference in nuclear EU signal intensity was observed between the LSDV-infected and mock groups (S4A and S4B Fig). In drug-treated groups, ENR, IDU, FIAU, RBV, and AraA markedly suppressed both cytoplasmic and nuclear EU signals (S4A–4C Fig). These findings indicate that ENR, IDU, FIAU, RBV, and AraA concurrently inhibit RNA synthesis of both LSDV and host cells at the indicated concentrations. At concentrations that effectively inhibited LSDV DNA synthesis, no significant suppression of host (S4D and S4E Fig) or LSDV (S4D and S4F Fig) RNA synthesis was observed with AraC treatment, and this lack of effect on host RNA synthesis persisted even with prolonged exposure (S4G and S4H Fig).

### AraC elicits marginal cytotoxicity regardless of LSDV infection

**Fig 6:**
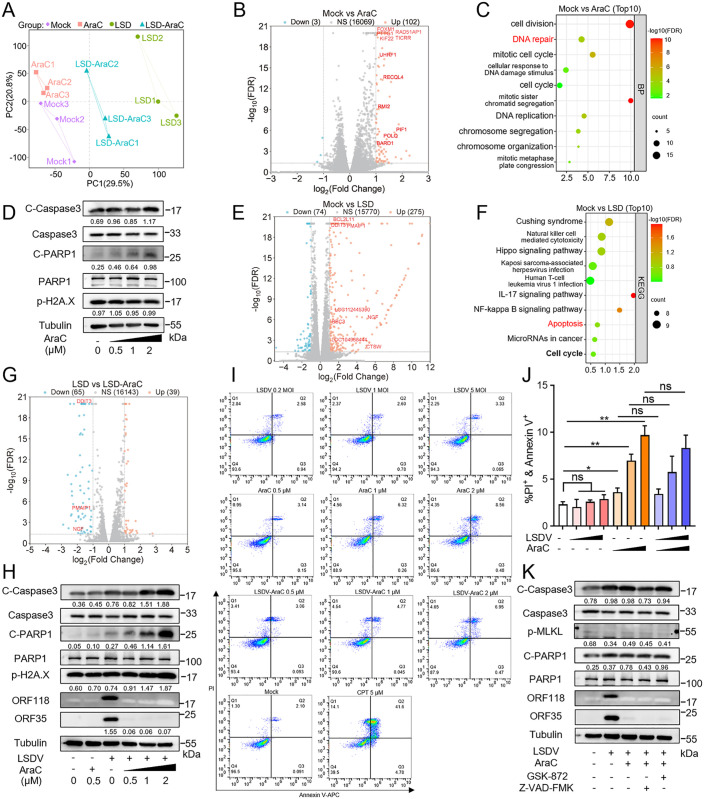
AraC elicits marginal cytotoxicity regardless of LSDV infection. (A) PC1-PC2 scatter plot from PCA of variance-stabilized gene-expression data. Each point denotes one biological replicate, color-coded by condition (Mock, AraC, LSD, and LSD-AraC). (B) Volcano plot of differentially expressed genes (DEGs) for Mock vs AraC(significance thresholds defined in Methods); significantly up- and down-regulated genes are highlighted. (C) GO term enrichment of DEGs from Mock vs AraC; top 10 terms (FDR < 0.05) ranked by the number of enriched DEGs. (D) MDBK cells were treated with AraC (0.5, 1, or 2 μM) for 48 h. Cell lysates were collected for western blotting using antibodies against cleaved PARP1 (C-terminus; also detects full-length PARP1), phospho-Histone H2A.X (Ser139), cleaved caspase-3 (also detects pro-caspase-3), and β-Tubulin. (E) Volcano plot of DEGs for Mock vs LSD. (F) KEGG pathway enrichment of DEGs from Mock vs LSD; top 12 pathways (FDR < 0.05) ranked by the number of enriched DEGs. (G) Volcano plot of DEGs for LSD vs LSD-AraC. (H) MDBK cells were mock-infected or infected with LSDV at an MOI of 1 for 48 h in the presence of 0, 0.5, 1, or 2 μM AraC. Cell lysates were harvested for western blotting with the indicated antibodies. (I and J) Cells subjected to the same treatments as in Figs 6D, 6H and S5A were stained with Annexin V-APC/PI and analyzed by flow cytometry (I), and the percentages of apoptotic cells in each group were statistically analyzed using GraphPad software (J). (K) MDBK cells were mock-infected or infected with LSDV at an MOI of 1 for 48 h with or without 0.5 μM AraC. The pan-caspase inhibitor Z-VAD-FMK (50 μM) and the RIPK3 inhibitor GSK872 (5 μM) were added to the indicated groups. Cell lysates were collected for western blotting with the indicated antibodies. The grayscale intensity of key protein bands in (D, H, and K) was normalized to the intensity of the internal control. Data are presented as mean ± SD, n = 3. *P < 0.05, **P < 0.01; ns, not significant.

Given that AraC mildly inhibits host DNA synthesis and may exert potential unintended cytotoxic effects, we performed RNA sequencing (RNA-seq) on AraC-treated cells, with or without LSDV infection, to delineate its impact on cellular fate from anti-viral effects. Differential expression analysis among the four treatment groups (Mock, AraC, LSD, and LSD-AraC) was used to assess the global impact of AraC on the host transcriptome. Principal component analysis (PCA) revealed clear separation of the four groups along PC1 (29.5%) and PC2 (20.8%), with tight clustering of biological replicates, indicating good reproducibility and that treatment conditions were the major determinants of transcriptional variance (Fig 6A). Notably, the LSD-AraC group showed the greatest separation from LSD infection alone, suggesting that AraC exerts broad transcriptional regulatory effects under viral infection.

Under non-infected conditions (Mock vs AraC), only 105 of the 16,174 quantified genes met the differential expression threshold (|log2FC| > 1, FDR < 0.05), accounting for just 0.65% of all genes (Fig 6B). Among these, 102 genes were upregulated and only 3 were downregulated. Gene Ontology (GO) enrichment identified 36 significantly enriched terms (FDR < 0.05), including 23 biological processes (BP), 7 cellular components (CC), and 6 molecular functions (MF). The upregulated BP terms were predominantly associated with DNA-related processes, such as cell division, DNA damage repair (DDR), cell-cycle regulation, and DNA replication, with all DNA repair-related genes being upregulated (Fig 6C). These findings indicate that AraC alone is sufficient to trigger host DNA damage response and repair pathways at the transcription level. We next validated these observations at the protein level by western blotting. Treatment of MDBK cells with 0.5-2 μM AraC resulted in concentration-dependent increases in the apoptosis markers cleaved PARP1 (C-PARP1) and cleaved Caspase-3 (C-Caspase3) (Fig 6D), whereas the double-stranded DNA break marker p-H2A.X (Ser139) did not show corresponding elevation. Meanwhile, the expression of full-length PARP1, a key mediator of DNA damage sensing and repair, remained relatively constant across conditions. Consistently, at the cellular level, Annexin V/PI staining revealed that AraC induced mild, dose-dependent apoptosis: early apoptotic cells remained below 1% at 0.5, 1, and 2 μM, while late apoptosis increased noticeably with higher doses (Fig 6I and 6J). These data suggest that at concentrations up to 2 μM (14-fold the IC_50_), AraC induces only limited DNA damage and apoptosis, and that the host DNA repair machinery may effectively buffer its cytotoxic effects, thereby keeping the overall apoptosis rate below 10%.

In the context of viral infection, RNA-seq analysis of the LSD group identified a total of 349 host differential expression genes (DEGs), including 275 upregulated and 74 downregulated genes (Fig 6E). KEGG enrichment revealed 37 significantly enriched pathways (*P* < 0.05), covering immune-related pathways such as IL-17 signaling, NF-κB, and TNF signaling, as well as apoptosis and cell-cycle regulation (Fig 6F). Consistent with these findings, western blot analysis showed that the levels of the apoptosis markers C-PARP1 and C-Caspase3 increased upon infection with higher multiplicity of infection (MOI) (S5A Fig). However, flow cytometry data (Fig 6I and 6J) demonstrated that LSDV infection at various MOIs did not enhance late apoptosis at the cellular level.

When AraC was added under viral infection (LSD vs LSD-AraC), the transcription of all viral genes was markedly reduced (S5B Fig). A total of 104 host DEGs were identified, including 39 upregulated and 65 downregulated genes (Fig 6G). KEGG analysis revealed 8 significantly enriched pathways, including necroptosis (S5C Fig), whereas apoptosis was not significantly enriched. GO analysis identified 7 enriched gene sets, largely associated with chromatin structure, nucleosome assembly, and DNA-related functions (S5D Fig), suggesting that the primary host effects of AraC involve DNA and chromatin regulation. Protein-level data further supported these observations: in the presence of LSDV infection, 0.5 μM AraC was sufficient to reduce viral early proteins to minimal levels and almost completely block viral late protein expression (Fig 6H). Although, AraC induced higher levels of C-PARP1 and C-Caspase3 under infection compared with either LSDV infection alone or AraC treatment alone (Fig 6H), AraC did not further enhance apoptosis during infection at the cellular level; instead, apoptosis levels were comparable to those induced by AraC alone (Fig 6I and 6J), indicating that AraC induced marginal cytotoxicity under infection conditions.

To further assess whether apoptosis or necroptosis contributes to the antiviral activity of AraC, specific pathway inhibitors were employed. Inhibition of apoptosis by the pan-caspase inhibitor Z-VAD-FMK did not rescue viral replication (Fig 6K), indicating that the antiviral effect of AraC is not mediated by apoptosis. Because KEGG analysis of the LSD vs LSD-AraC comparison suggested a potential involvement of necroptosis, we examined the phosphorylation of MLKL (a key necroptosis effector). p-MLKL levels remained low across all treatment groups, indicating that AraC does not induce necroptosis; accordingly, inhibiting necroptosis using GSK-872 failed to restore viral protein expression (Fig 6K). Collectively, these results demonstrate that the antiviral activity of AraC primarily derives from its inhibition of viral DNA synthesis rather than engagement of apoptotic or necroptotic cytotoxicity.

### AraC blocks the transcription of viral late genes

It has been reported that the replication cycle of orthopoxviruses can be divided into early and late phases according to the temporal cascade of gene expression, with the late phase characterized by structural protein expression and progeny virion assembly; viral DNA synthesis is a prerequisite for late gene transcription [27]. To investigate how AraC affects the LSDV replication cycle, MDBK cells were infected at an extreme high MOI of 10 to synchronize viral replication and allow monitoring within a single replication cycle. AraC was added during infection to inhibit viral DNA synthesis, and the progression of the viral replication cycle was subsequently assessed by examining the expression of viral early and late genes.

**Fig 7:**
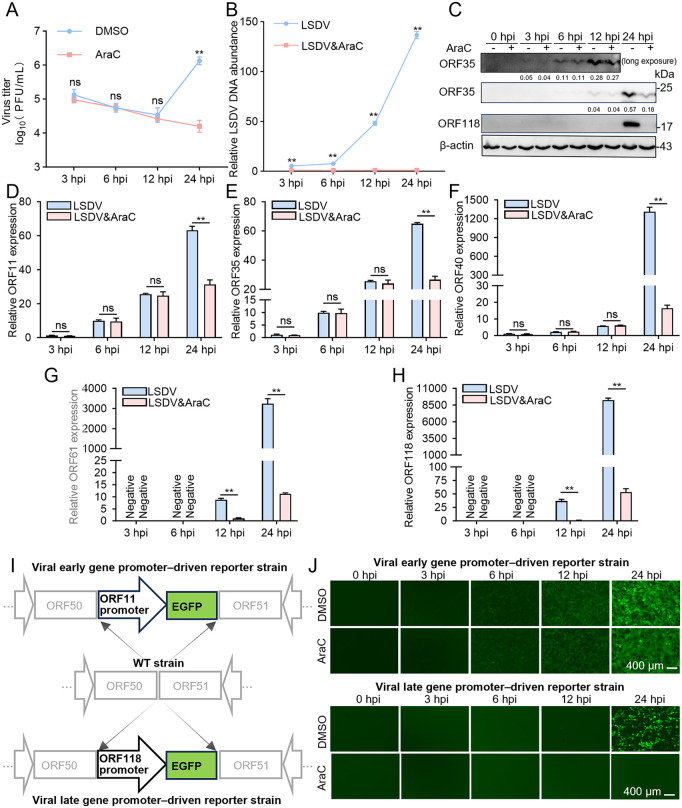
AraC blocks the transcription of viral late genes. (A-F) MDBK cells were infected with LSDV at an MOI of 10 and treated with DMSO or 0.5 μM AraC. Samples were collected at the indicated times and analyzed as follows: (A) virus titers determined by plaque assay; (B) viral DNA quantified by qPCR and normalized to host *β-actin*; (C) western blotting of viral proteins *ORF35* (early) and *ORF118* (late) with actin as a loading control; (D-H) RT-qPCR analysis of *ORF11* (D), *ORF35* (E), *ORF40* (F), *ORF61* (G), and *ORF118* (H), normalized to actin mRNA. (I) Schematic illustrating the construction of early- and late- gene promoter driven reporter viruses. (J) MDBK cells were infected with the early gene promoter-driven reporter virus (top) or the late gene promoter-driven reporter virus (bottom) at an MOI of 10 and treated with DMSO or 0.5 μM AraC. EGFP fluorescence images were acquired at the indicated time points. The grayscale intensity of key protein bands in (C) was normalized to the intensity of the internal control. Scale bars are shown in the lower right corner in (J). Data are presented as mean ± SD, n = 3. **P < 0.01; ns, not significant.

With respect to viral growth, virus titers in the DMSO control changed little before 12 hpi but rose markedly at 24 hpi, whereas titers in the AraC-treated group did not increase throughout the observation period (Fig 7A). These data indicate that the first replication cycle was completed between 12 and 24 hpi and that AraC effectively blocked this process. Consistently, quantification of viral DNA showed no accumulation in the AraC group, while the DMSO group exhibited a time-dependent increase from 3 to 24 hpi (Fig 7B).

At the transcription level, western blot detected the viral early protein ORF35 as early as 3 hpi, with its abundance between 3 and 12 hpi largely unaffected by AraC (Fig 7C); by contrast, the viral late protein ORF118 was detected only at 24 hpi in the DMSO group, indicating that AraC markedly suppressed its protein expression (Fig 7C). Concordantly, qPCR revealed that transcripts of early genes (*ORF11, ORF35, ORF40*) were detectable at 3 hpi and were not significantly affected by AraC from 3 to 12 hpi (Fig 7D–7F), with differences emerging at 24 hpi; in contrast, transcripts of late genes (*ORF61, ORF118*) first appeared at 12 hpi and were significantly reduced by AraC at both 12 and 24 hpi (Fig 7G and 7H).

To further validate these findings, we constructed EGFP reporter viruses driven by the LSDV *ORF11*- or *ORF118*- promoter (Fig 7I). Under high-MOI infection, *ORF11* promoter-driven EGFP signals were visible by 3 hpi and showed little change through 12 hpi, whereas *ORF118* promoter-driven EGFP was observed at 12 hpi only in the DMSO group and was absent in the AraC group (Fig 7J). Collectively, these results indicate that AraC has limited effects on early transcription but robustly suppresses late gene promoter activity and expression; together with the DNA quantification and viral titer data, we infer that AraC inhibits viral DNA synthesis and arrests the LSDV replication program prior to the late phase.

### Structural basis underlying AraC inhibition of LSDV DNA polymerase

**Fig 8:**
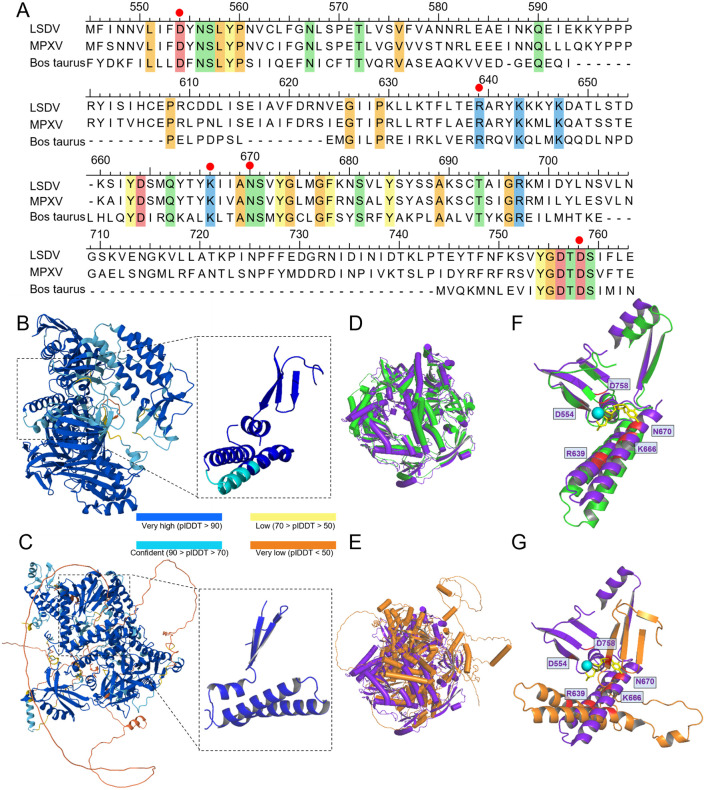
Structural basis of AraC-mediated inhibition of LSDV replication through targeting of viral DNA polymerase. (A) Multiple sequence alignment of DNA polymerases from LSDV, bovine, and MPXV was performed, and key residues involved in AraC binding to MPXV DNA polymerase are highlighted with red solid circles. (B and C) Predicted three-dimensional structures of LSDV DNA polymerase (B) and bovine DNA polymerase α subunit (C) were predicted using AlphaFold3. (D and E) Structural alignment of MPXV DNA polymerase (purple, PDB: 8K8S) with predicted LSDV DNA polymerase (green, D) or predicted bovine DNA polymerase α subunit (brown, E) was performed; (F and G) Three-dimensional structural alignment of MPXV DNA polymerase with predicted LSDV DNA polymerase (F) or predicted bovine DNA polymerase α subunit (G) in the AraC binding pocket region was performed; amino acid residues involved in Ara-CTP binding are highlighted in red, and Ara-CTP is shown as yellow.

The mechanism by which AraC inhibits the DNA polymerase of the MPXV has recently been elucidated at the protein structural perspective. Ara-CTP, the active metabolite of AraC, forms an additional hydrogen bond with N665 near the deoxycytidine triphosphate (dCTP) binding site, which enhances its binding affinity compared to dCTP, thereby terminating DNA synthesis [28,29]. To analyze the inhibitory mechanism of AraC on LSDV DNA polymerase (NCBI: XNX23144.1), we compared the amino acid sequence conservation among LSDV DNA polymerase, bovine DNA polymerase α subunit (NCBI: NP_001192994), and MPXV DNA polymerase. The results revealed that the amino acid binding sites (D549, R634, K661, N665, D753) of MPXV DNA polymerase were fully conserved in LSDV DNA polymerase (D554, R639, K666, N670, D758) and bovine DNA polymerase α subunit (D860, R922, K950, N954, D1004) (Fig 8A).

Further homology modeling of LSDV DNA polymerase and bovine DNA polymerase α subunit was performed using AlphaFold3 [30]. The predicted structures exhibited predicted local distance difference test (pLDDT) score greater than 90 for all conserved sites, indicating very high reliability (Fig 8B and 8C). Structural alignment of the modeled LSDV DNA polymerase and bovine DNA polymerase α subunit with the crystal structure of MPXV DNA polymerase demonstrated that the overall three-dimensional structure of LSDV DNA polymerase closely resembled that of MPXV DNA polymerase (Fig 8D), with similar conformations of the Ara-CTP binding pocket region (Fig 8F). In contrast, the bovine DNA polymerase α subunit displayed significant overall structural divergence from MPXV DNA polymerase (Fig 8E), although the conformations of the Ara-CTP binding pocket region remained similar (Fig 8G). These findings suggest that the mechanism by which Ara-CTP inhibits the activity of LSDV DNA polymerase may be achieved by binding to D554, R639, K666, N670, D758 of LSDV DNA polymerase to suppress DNA synthesis.

## Discussion

The recent emergence of LSDV has raised global concerns [31]. Originally endemic to Africa, this pathogen has now spread to Europe and Asia, posing a serious threat to the global cattle industry [32]. The limited fundamental research on LSDV, coupled with the absence of antiviral drugs, often results in complete economic devaluation of diseased cattle. In response to the urgent epidemic situation, we established a dual-reporter recombinant LSDV system expressing both mCherry and firefly luciferase under the poxvirus early/late H5 promoter [33]. This platform retained replication characteristics equivalent to the parental virus, enabled real-time visualization of infection dynamics, and provided highly sensitive quantitative readouts. Compared with EGFP-based systems [34], the dual-reporter design substantially improved signal-to-noise ratio, assay accuracy, and screening throughput, effectively accelerating LSDV drug discovery.

**Fig 9:**
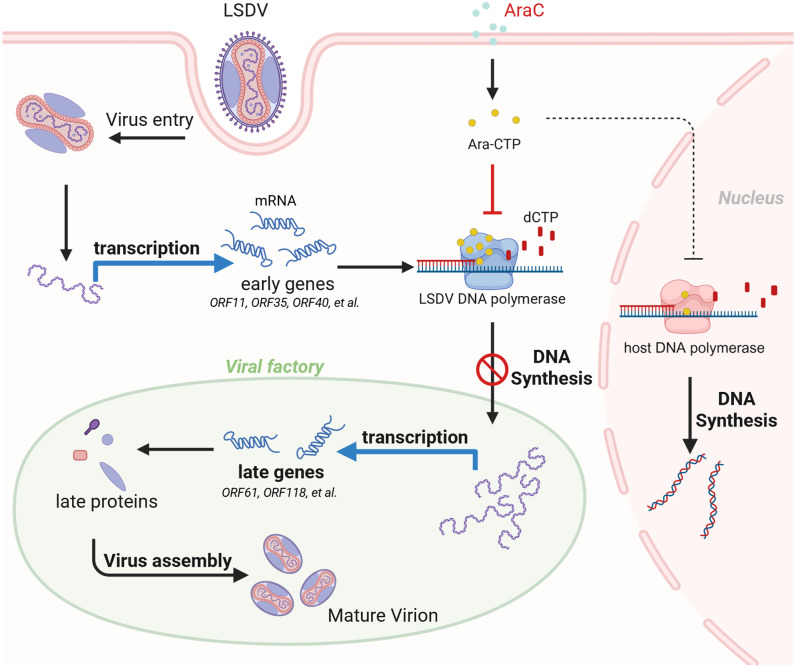
Schematic diagram illustrating AraC inhibiting LSDV replication. During LSDV infection, AraC targets the viral DNA polymerase to inhibit viral DNA synthesis, thereby preventing the activation of late-gene transcription and ultimately blocking progression of the replication cycle from the early to the late phase. This figure was created with BioRender.com. Created in BioRender. Gong, Z. (2026) https://BioRender.com/7ugrs73.

Through this platform, we identified six compounds with significant anti-LSDV activity, five of which were nucleotide analogs (IDU, FIAU, RBV, AraC, and AraA), using a drug repurposing strategy. This strong enrichment of nucleoside analogs highlights viral nucleic acid polymerases as key therapeutic targets for LSDV. Similar to other large DNA viruses, LSDV encodes its own DNA polymerase to mediate genome replication. Our findings show that AraC potently suppresses viral DNA synthesis, nearly abolishing late viral gene expression while sparing early gene transcription. This suggests that the synthesis and accumulation of viral DNA is not merely a genomic requirement but also a prerequisite for the activation of late gene transcription, and that disruption of this step effectively prevents virion assembly and the establishment of productive infection (Fig 9).

Notably, the SI of AraC varied between MDBK and Vero cells, likely reflecting species-dependent differences in nucleoside metabolism and transport. AraC requires phosphorylation by deoxycytidine kinase (dCK) to become the active metabolite Ara-CTP [35], while being inactivated by cytidine deaminase (CDA) and NT5C2 [36]. Variation in dCK/CDA/NT5C2 activity, nucleoside transporter levels [35], nucleotide pool homeostasis [37,38], and polymerase sensitivity [39] may together shape the antiviral and cytotoxic profiles across host species.

Structural prediction further indicates that Ara-CTP may interact with the conserved catalytic residues of the LSDV DNA polymerase (D554, R639, K666, N670, and D758) in a manner similar to that observed for the MPXV polymerase, potentially forming an additional hydrogen bond with Asn670 that enhances binding affinity while preventing subsequent chain elongation. Notably, this structural basis is highly conserved not only in bovine-derived DNA polymerase but also among other members of the Capripoxvirus genus, suggesting that AraC may exert broad-spectrum inhibitory activity against capripoxviruses. On the other hand, such structural conservation may also underlie the potential of AraC to partially inhibit host DNA synthesis. Nevertheless, transcriptomic profiling showed that AraC did not induce broad host gene dysregulation; only 105 genes were altered in uninfected cells, mostly linked to DNA damage response and cell cycle pathways. Although C-PARP1 and C-Caspase 3 increased mildly, late apoptosis and p-H2AX levels remained low, indicating minimal DNA break accumulation. Functional assays further confirmed that AraC’s antiviral activity is independent of apoptosis or necroptosis, as caspase inhibition or necroptosis blockade failed to restore viral replication. Given that replication-stress–associated DNA damage is a major driver of AraC cytotoxicity [40], the absence of strong apoptotic or necroptotic activation within the antiviral window highlights its favorable safety profile and supports a model in which AraC acts primarily through inhibition of viral DNA synthesis.

A notable finding is the divergent recovery of DNA synthesis between host and virus under prolonged AraC exposure. Viral DNA synthesis remained completely suppressed, whereas host chromosomal replication gradually recovered. This asymmetry may be attributed to the robust nuclear DNA repair machinery in host cells, enabling resolution of AraC-induced replication stress. In contrast, LSDV replicates exclusively within cytoplasmic viral factories and lacks a functionally characterized DNA repair system, leaving its genome far more susceptible to irreversible chain termination. These mechanistic distinctions confer AraC with an intrinsic advantage for selective interference with viral DNA replication over host DNA replication.

The candidate compounds identified through drug repurposing strategy demonstrate significant safety advantages. Notably, IDU [41], RBV [42], Ara-C [43], and Ara-A [44] have already been approved for clinical treatment of human diseases, which enhances confidence in their further development as therapeutic agents for LSDV in cattle. Additionally, ENR has been widely employed in animal husbandry to treat and prevent bacterial infections, particularly bovine mycoplasma [45]. Given its safety profile in cattle, its repurposing as an anti-LSDV therapeutic agent presents significant advantages. However, our experimental results indicate that while these nucleotide analogs effectively inhibit LSDV nucleic acid synthesis, they also exert some influence on host nucleic acid synthesis. Therefore, future work should focus on optimizing dosing regimens and delivery strategies, as well as developing next-generation analogs with enhanced viral selectivity to minimize host-associated effects.

In summary, this study successfully established a high-throughput drug screening platform for LSDV, and identified six compounds with significant anti-LSDV activity. These compounds primarily function during the replication phase by targeting viral nucleic acid synthesis to effectively inhibit LSDV replication. Given their established safety profiles and potent antiviral efficacy, these compounds hold substantial developmental value and warrant further investigation as promising candidates for treating LSDV infections.

## Materials and methods

### Cells, virus and antibodies

The MDBK (ATCC CCL-22), Vero (ATCC CCL-81) and BHK-21 (ATCC CCL-10) cell lines were purchased from ATCC and maintained in Dulbecco’s Modified Eagle Medium (DMEM, Servicebio, China) supplemented with 10% fetal bovine serum (FBS, ExCell, China), 100 U/mL penicillin, and 100 μg/mL streptomycin (YEASEN, China). The LSDV XJ201901 strain (GenBank: OM984485), together with the LSDV rabbit polyclonal antibody, the LSDV ORF35 mouse polyclonal antibody, and the LSDV ORF118 mouse monoclonal antibody, was maintained in our laboratory. The C-Caspase3, C-PARP1 and p-MLKL (S345) were purchased from HUABIO (China); p-Histone H2A.X (S139) were purchased from Beyotime (China); The β-actin, β-tubulin mouse monoclonal antibody, and HRP-labeled goat anti-mouse and goat anti-rabbit secondary antibody were purchased from Epizyme (China). Alexa Fluor 594 labeled goat anti-rabbit IgG (H + L) was purchased from Invitrogen (USA).

### Construction of recombinant virus

CRISPR-Cas9 technology can be used to improve the efficiency of poxvirus modification [46,47]. Since LSDV strictly replicates in the cytoplasm, we constructed a dual-sgRNA-expressing rPX458 plasmid (rPX458-sgRNA1 + 2), in which the Cas9 was modified by removing its NLS to ensure its cytoplasmic localization (Fig 1A) [46,47]. Specifically, the PX458 plasmid (Addgene: #48138) was used as a template to design three primer pairs (P1F/P1R, P2F/P2R and P3F/P3R in S1 Table). PCR amplification was performed using the PX458 plasmid as a template, and the purified PCR products were ligated via seamless cloning kit (Beyotime, China) to remove the nuclear localization signal (NLS) sequences flanking the Cas9, resulting in the modified rPX458 plasmid. Then, using the CRISPOR online tool [48], two pairs of specific oligo sequences were designed targeting the regions near *ORF50* (P4F/ P4R) and *ORF51* (P5F/ P5R in S1 Table) of the LSDV. Each oligo pair was annealed to form double-stranded DNA fragments with sticky ends. The rPX458 plasmid was digested with BbsⅠ (Thermo Fisher, USA), and the digested product was ligated with the annealed oligos using T4 DNA ligase (YEASEN, China) to construct the rPX458-sgRNA1 and rPX458-sgRNA2 plasmids, respectively. Subsequently, the rPX458-sgRNA1 and rPX458-sgRNA2 plasmids were linearized by digestion with KpnⅠ, and the sgRNA2 region of rPX458-sgRNA2 was amplified using primers (P6F/P6R). The amplified product was then ligated with the linearized rPX458-sgRNA1 plasmid using a seamless cloning kit, yielding the final rPX458-sgRNA1 + 2 dual sgRNA plasmid.

According to reports, the insertion of foreign genes between *ORF50* and *ORF51* in the genome of LSDV does not affect the viral replication efficiency [49]. Therefore, we chose the intergenic region between these two genes as the insertion site, and a donor plasmid was constructed (Fig 1A). In detail, the left homology arm (P7F/P7R) and right homology arm (P8F/P8R) were amplified by PCR using LSDV genomic DNA as a template. Meanwhile, the H5 promoter-driven mCherry and luciferase expression cassette was synthesized by Tsingke (China) and amplified using primers (P9F/P9R). Additionally, the backbone region of the pVAX1 plasmid (Thermo Fisher, USA) was amplified using primers (P10F/P10R). Finally, these four fragments were assembled using a seamless cloning kit to construct the rLSDV donor plasmid. Using the same strategy, donor plasmids for the *ORF11* promoter-EGFP strain and the *ORF118* promoter-EGFP strain were constructed. The only difference from the rLSDV donor plasmid was the expression cassette inserted between the *ORF50* and *ORF51* genes. Specifically, an EGFP expression cassette driven by the *ORF11* promoter (150 bp upstream of the *ORF11* start codon, ATG) was synthesized by Tsingke Biotechnology (China) and amplified using primers P11F/P11R in S1 Table. Likewise, an EGFP expression cassette driven by the *ORF118* promoter (182 bp upstream of the *ORF118* start codon) was synthesized by Tsingke Biotechnology (China) and amplified using primers P12F/P11R in S1 Table. The amplified fragments were then inserted into the corresponding donor plasmids via homologous recombination, resulting in the construction of the ORF11-EGFP and ORF118-EGFP donor plasmids. All PCR and restriction enzyme digestion products were purified using a DNA purification kit (YEASEN, China) prior to ligation. The recombinant plasmids were verified by Sanger sequencing and subsequently transformed into E. coli DH5α for amplification. Plasmid extraction was performed using an endotoxin-free plasmid extraction kit (TIANGEN, China) for downstream applications.

Using the above-mentioned plasmids, the viral genome was modified according to the schematic illustration (Fig 1B). Briefly, BHK-21 cells were co-transfected with the donor plasmid and rPX458-sgRNA1 + 2 plasmid using Lipofectamine 3000 (Thermo Fisher, USA). At 12 hours post-transfection, cells were infected with WT LSDV at a MOI of 1. The cells were harvested at 72 hpi, subjected to three freeze-thaw cycles, and the lysates were used to inoculate MDBK cells for 2 h. The supernatant was then replaced with maintenance medium (DMEM supplemented with 2% FBS, 100 U/mL penicillin, and 100 μg/mL streptomycin) containing 1% methylcellulose (Beyotime, China). At 72 hpi, fluorescent plaques were selected under a fluorescence microscope for the next round of purification. Three rounds of purification were performed, following which genomic DNA was extracted from the purified virus for PCR validation and sanger sequencing to confirm the intended genetic modifications.

### Western blot

The cells were lysed with lysis buffer (50 mM Tris, 150 mM NaCl, 1% Triton X-100, 1% sodium deoxycholate, 0.1% SDS, pH 7.4) for 15 min on ice. After centrifugation, the supernatant containing cell lysates was collected and denatured with loading buffer by boiling at 95°C for 5 min. The protein samples were separated by SDS-PAGE electrophoresis and subsequently transferred onto a nitrocellulose (NC) membrane (Cytiva, USA). The membrane was blocked with 5% skim milk and then incubated with the corresponding primary antibodies and secondary antibody. Protein bands were visualized using Super ECL Detection Reagent (Yeasen, China) with an Automated Chemiluminescence Imaging System (Tanon, China). For proteins with overlapping or conflicting molecular weights, membranes were stripped using antibody stripping buffer (Cwbio, China) to remove both primary and secondary antibodies. After restripping, membranes were re-blocked and incubated with a different set of primary and secondary antibodies for subsequent detection.

### qPCR

At the indicated time points, cells were collected, and genomic DNA was extracted using a Cell/Tissue DNA Extraction Kit (YEASEN, China) according to the manufacturer’s instructions. The relative abundance of viral DNA was quantified by quantitative real-time PCR (qPCR) on a QuantStudio 6 Real-Time PCR System (Applied Biosystems, USA) using SYBR Green qPCR Master Mix (YEASEN, China). Specific primers targeting the LSDV *ORF72* gene (S1 Table) were used for amplification. The relative viral DNA abundance was calculated using the 2^(-ΔΔCt) method, normalized to the abundance of *β-actin* as an internal reference.

For analysis of host gene mRNA expression, total RNA was extracted from cells at the indicated time points using RNAiso Plus reagent (Takara, Japan) according to the manufacturer’s instructions. cDNA was synthesized using the Hifair II 1st Strand cDNA Synthesis SuperMix (YEASEN, China). Relative mRNA expression levels were determined by qPCR using gene-specific primers (S1 Table) and calculated by the 2^(-ΔΔCt) method, normalized to Bos taurus *β-actin* expression.

### Viral titer determination

MDBK cells were seeded in 24-well plates and infected with LSDV. At 72 hpi, the cells were harvested through three freeze-thaw cycles. Following centrifugation at 4,000 × g for 20 min at 4°C, the supernatant was collected. MDBK cell monolayers in 48-well plates were then infected with serially diluted virus supernatant for 2 h. The inoculum was subsequently replaced with fresh maintenance medium containing 1% methylcellulose. At 120 hpi, viral fluorescent plaque forming units (PFU) were quantified by immunofluorescence assay (IFA). Briefly, the medium was aspirated and cells were fixed with 4% paraformaldehyde for 20 min. Fixed cells were permeabilized with PBS containing 0.3% Triton X-100 for 20 min, followed by blocking with 3% BSA for 1 h. Immunostaining was performed by sequential incubation with ORF118 mouse monoclonal antibody and Alexa Fluor 488-conjugated goat anti-mouse secondary antibody (1:3,000 dilution), each for 1 h at room temperature. PFU were determined by fluorescence microscopy.

### Half-maximum cytotoxic concentration (CC_50_)

Cell viability was assessed using CCK-8 assays, with drugs tested at eight concentrations via three-fold serial dilution series starting at 100 μM. 200 μL of serial concentrations of the test drugs were added to a 96-well plate containing a monolayer of MDBK or Vero cells. The plates were incubated for 72 h. 10 μL of CCK-8 in 100 μL maintenance medium was added to each well. The plates were incubated for 2 h. To calculate the CC_50_, the absorbance at 450 nm was measured and normalized to the DMSO-treated group. The cytotoxicity curves and CC_50_ values were generated using GraphPad Prism 7.0 (GraphPad Software, USA).

### Half-maximal inhibitory concentration (IC_50_)

Confluent monolayers of MDBK or Vero cells in 96-well plates were treated with 30 μL of serially diluted test drugs, followed by infection with rLSDV (0.1 MOI). After a 2 h adsorption period, 170 μL of additional drug dilutions were added. The plates were then incubated for 72 h. For IC_50_ determination, luciferase activity was measured in relative light unit (RLU) using a Luciferase Reporter Gene Assay Kit (YEASEN, China) following the manufacturer’s instructions. Viral replication levels were reflected by the ratio of luciferase activity in drug-treated groups to DMSO-treated groups. Dose-inhibition curves and IC_50_ values were generated using GraphPad Prism 7.0.

### Time-of-addition assay

To delineate the antiviral mechanism, we performed time-of-addition experiments to identify the specific LSDV life cycle stages targeted by the drug. Direct inactivation of virus (Virucidal): The drug was mixed with the virus and incubated at 37°C for 1 h, followed by infection of MDBK cells for 2 h to assess whether the drug directly inactivates viral particles. Cell receptor antagonism (Pre): Cells were pretreated with the drug at 37°C for 2 h, after which the drug was removed, and the cells were infected with the virus for 2 h to determine whether the drug inhibits viral attachment by blocking cellular receptors. Viral Fusion/Entry Inhibition (During): The virus and drug were added to cells simultaneously and co-incubated for 2 h to examine whether the drug interferes with viral fusion or internalization. Viral replication inhibition (Post): Cells were first infected with the virus for 2 h, after which the drug was added to the culture medium to evaluate its effect on viral replication. DMSO-treated groups served as controls in all four experimental conditions. Following viral infection, cells in each group were maintained for an additional 72 h. At 72 hpi, mCherry fluorescence was observed under a fluorescence microscope, and samples were collected to measure luciferase activity.

### Assessment of DNA or RNA synthesis

MDBK cells were seeded in confocal dishes and cultured for 24 h, followed by infection with LSDV (1 MOI). At 24 hpi, the cells were pretreated with the specified concentrations of drugs for 60 min. Subsequently, 5-ethynyl-2’-deoxyuridine (EdU) was added at a final concentration of 10 μM, and the cells were further incubated for the indicated time to allow EdU incorporation into newly synthesized DNA. According to the manufacturer’s instructions of the BeyoClick EdU Cell Proliferation Kit (Beyotime, China), the click reaction working solution was prepared, and EdU was labeled with Alexa Fluor 488. Finally, the total DNA were stained with Hoechst 33342 for 10 min. Throughout all procedures, the cells were washed three times with PBS after each solution change. The fluorescence was observed using a confocal microscope (Nikon A1 plus, Japan). The same procedure was followed to assess the effect of the drugs on LSDV RNA synthesis using the BeyoClick EU Cell Proliferation Kit (Beyotime, China), with the 5-ethynyl uridine (EU) working concentration adjusted to 100 μM.

### RNA-seq

Total RNA was extracted from MDBK cells under four experimental conditions: Mock (uninfected control), AraC (0.5 μM AraC treatment), LSD (infection with LSDV at 1 MOI), and LSD-AraC (infection with LSDV at 1 MOI in the presence of 0.5 μM AraC). Each condition included three biological replicates. Cells were harvested at 48 hpi for RNA extraction using RNAiso Plus reagent. RNA quality and integrity were assessed using an Agilent 2100 Bioanalyzer (Agilent, USA), and samples with RNA Integrity Number (RIN) ≥ 8.0 were selected for library construction.

Sequencing libraries were prepared using the Illumina TruSeq RNA Sample Preparation Kit following the manufacturer’s instructions and sequenced on an Illumina platform (paired-end, 150 bp read length). Raw reads were quality-checked and trimmed using fastp (v0.23.2) to remove adapter sequences and low-quality bases. Clean reads were then aligned to both the Bos taurus reference genome (GCF_002263795.3) and the LSDV genome (GCA_024266845.1) using HISAT2 (v2.2.1). The dual mapping strategy enabled simultaneous quantification of host and viral transcripts.

Gene expression levels were quantified as fragments per kilobase of transcript per million mapped reads (FPKM) using StringTie (v2.2.1). Differentially expressed genes (DEGs) between groups were identified with DESeq2 (v1.38.0) using the criteria log₂(fold change) ≥ 1 and false discovery rate (FDR) < 0.05. Functional enrichment analyses, including Gene Ontology (GO) and Kyoto Encyclopedia of Genes and Genomes (KEGG) pathway analyses, were conducted using the clusterProfiler package (v4.6.0). All statistical analyses and data visualization were performed in R (v4.3.1).

### Flow cytometric (FC) analysis of cell apoptosis

MDBK cells were infected with LSDV or mock-infected and treated with or without AraC at the indicated final concentrations. A CPT-treated group was included as a positive control for apoptosis induction. At 48 hpi, cell culture supernatants were collected and centrifuged at 1000 rpm for 10 min at 4°C to harvest non-adherent apoptotic cells. Adherent cells were detached using trypsinization and combined with the pelleted cells from the supernatant. The collected cells were washed once with pre-chilled PBS (4°C), and apoptosis was assessed using the Annexin V-APC/PI Apoptosis Kit (Elabscience, China) according to the manufacturer’s instructions. Samples were analyzed immediately using a BD Accuri C6 flow cytometer (Becton Dickinson, USA).

### Transmission electron microscopy (TEM)

MDBK cells infected with the WT LSDV or rLSDV were fixed with pre-chilled 2.5% glutaraldehyde solution (Yuanye, China), and harvested using a cell scraper. Subsequently, the fixed samples were processed into ultrathin sections, and viral particles were visualized and imaged using a HITACHI HT7800 transmission electron microscope (Hitachi, Japan).

### Cell viability assay

MDBK and Vero cells were seeded in 96-well plates and treated with varying concentrations of drugs (MedChemExpress, USA). After 72 h of treatment, 100 μL of DMEM medium containing 10 μL CCK-8 (Vazyme, China) was added to each well, followed by incubation at 37°C for 2 h. The absorbance was then measured at OD450 nm using a microplate reader.

### Statistical analysis

All experiments were performed at least three times, and results are expressed as mean ± SD. Data were analyzed using GraphPad Prism software (version 7.0), and statistical significance between groups was determined using Student’s t-test or one-way ANOVA. Differences were considered statistically significant at *P* < 0.05 (*), *P* < 0.01 (**); “ns” indicates no significant difference.

## Supporting information

S1 Fig CC_50_ or IC_50_ of the drugs. Cells were treated with 3-fold serially diluted drugs for 72 h; cell viability was determined by CCK-8 assay, and dose-inhibition curves were generated using GraphPad Prism 7 to calculate CC_50_. rLSDV-infected (0.1 MOI) cells were treated with three-fold serially diluted enrofoxacin (monohydrochloride) for 72 h, and viral replication was assessed by luciferase activity; dose-inhibition curves were generated using GraphPad Prism 7 to calculate IC_50_. Data are expressed as means ± SD, n = 3. The data are representative of results from three independent experiments.
(S1_Fig.TIF)

S2 Fig IDU, FIAU, and Ara-A inhibit the DNA synthesis of LSDV. (A) MDBK cells were infected with LSDV (1 MOI). At 24 hpi, the cells were treated with ENR (100 μM), IDU (20 μM), FIAU (5 μM), RBV (20 μM), or Ara-A (20 μM) for 1 h, followed by labeling of newly synthesized DNA with EdU for 1 h. Viral and host DNA synthesis was then assessed by fluorescence microscopy. (B and C) The relative fluorescence units (RFU) of EdU in the nucleus (B) and cytoplasm (C) were quantified using Image J software. Scale bars are shown in the lower right corner in (A). Data are presented as mean ± SD, n = 3. **P < 0.01; ns, not significant.
(S2_Fig.TIF)

S3 Fig The distribution of newly synthesized RNA in MDBK cells at different time points after EU addition. EU was added at a final concentration of 100 μM to MDBK cells, and the distribution of RNA in the cells was detected by laser confocal microscopy at 10 min, 20 min, 30 min, 45 min and 60 min after addition, respectively. The data are representative of results from three independent experiments.
(S3_Fig.TIF)

S4 Fig Anti-LSDV candidates suppress viral RNA synthesis, with the exception of AraC, at effective antiviral levels. (A-C) MDBK cells were infected with LSDV at 1 MOI. At 24 hpi, cells were treated with ENR (100 μM), IDU (20 μM), FIAU (5 μM), RBV (20 μM), or AraA (20 μM) for 1 h, followed by an additional 1 h incubation with the same compounds in the continued presence of EU to label newly synthesized RNA (A). Viral proteins were detected using rabbit anti-LSDV polyclonal antibodies. Nuclear (B) and cytoplasmic (C) RFU values were quantified using ImageJ. (D-F) MDBK cells were infected with LSDV at 1 MOI. At 24 hpi, cells were treated with AraC (0.5 μM) for 1 h, followed by an additional 1 h incubation with AraC (0.5 μM) in the continued presence of EU to label newly synthesized RNA (D). Viral proteins were detected using rabbit anti-LSDV polyclonal antibodies. Nuclear (E) and cytoplasmic (F) RFU values were quantified using ImageJ. (G and H) MDBK cells were treated with AraC (0.5 μM) for 1 h, followed by incubation for the indicated times with AraC (0.5 μM) in the continued presence of EU to label newly synthesized RNA (G). The cytoplasm and plasma membrane were stained with DiD. RFU values were quantified using ImageJ. Scale bars are shown in the lower right corner in (A, D and G). Data are presented as mean ± SD, n = 3. *P < 0.05; **P < 0.01; ns, not significant.
(S4_Fig.TIF)

S5 Fig Supplementary figures supporting the RNA-seq analysis of the host transcriptomic response to AraC during LSDV infection. (A) MDBK cells were mock-infected or infected with LSDV at an MOI of 0.2,1 and 5 for 48 h; cell lysates were harvested for western blotting with the indicated antibodies. (B) Heatmap showing the fold-change differences of all viral ORF genes from LSD vs LSD-AraC in the RNA-seq experiment. (C and D) KEGG pathway and GO enrichment of DEGs from LSD vs LSD-AraC.
(S5_Fig.TIF)

S1 Table Primers used in this study. (S1_Table.XLSX)
