# Supplementary figures and images for: Anti-orthopoxvirus drugs inhibit lumpy skin disease virus replication by targeting viral DNA polymerase
Source: PLoS Pathog. 2026 Jan 26;22(1):e1013903. doi: 10.1371/journal.ppat.1013903 (PMC12858068; doi:10.1371/journal.ppat.1013903)

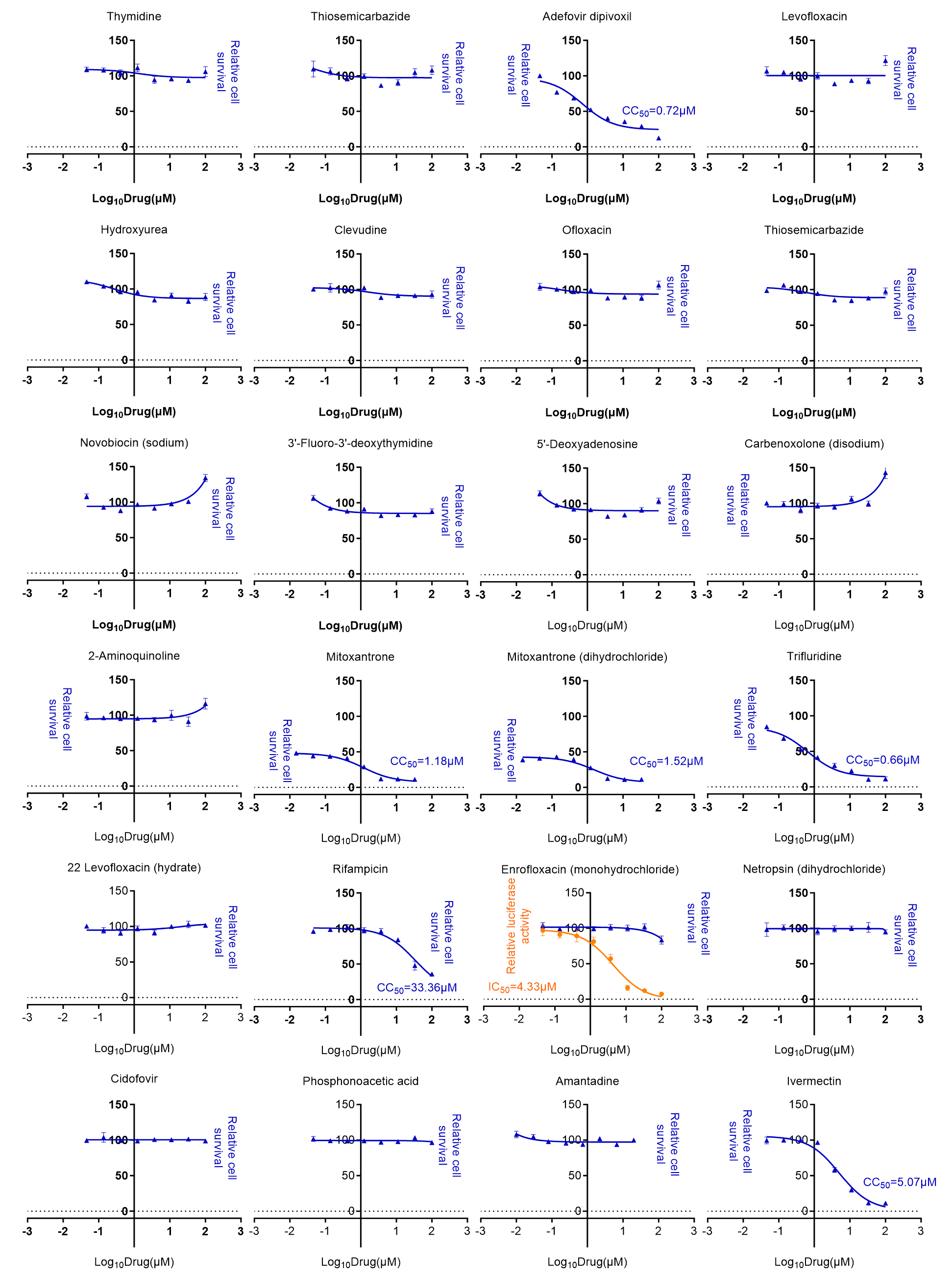

Supplement: S1 Fig — Cells were treated with 3-fold serially diluted drugs for 72 h; cell viability was determined by CCK-8 assay, and dose-inhibition curves were generated using GraphPad Prism 7 to calculate CC50. rLSDV-infected (0.1 MOI) cells were treated with three-fold serially diluted enrofoxacin (monohydrochloride) for 72 h, and viral replication was assessed by luciferase activity; dose-inhibition curves were generated using GraphPad Prism 7 to calculate IC50. Data are expressed as means ± SD, n = 3. The data are representative of results from three independent experiments. (S1_Fig.TIF) [file ppat.1013903.s001.tif]

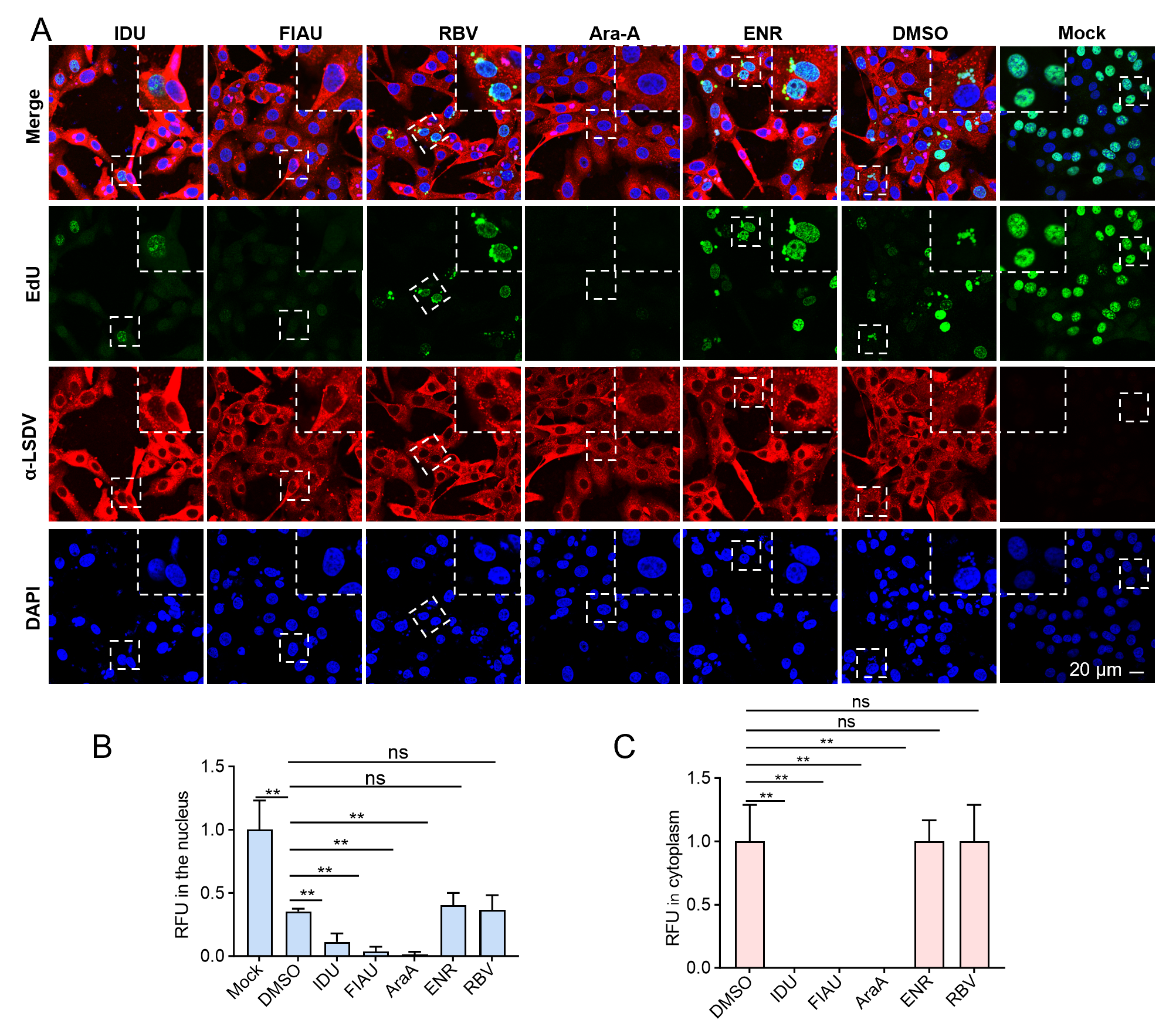

Supplement: S2 Fig — (A) MDBK cells were infected with LSDV (1 MOI). At 24 hpi, the cells were treated with ENR (100 μM), IDU (20 μM), FIAU (5 μM), RBV (20 μM), or Ara-A (20 μM) for 1 h, followed by labeling of newly synthesized DNA with EdU for 1 h. Viral and host DNA synthesis was then assessed by fluorescence microscopy. (B and C) The relative fluorescence units (RFU) of EdU in the nucleus (B) and cytoplasm (C) were quantified using Image J software. Scale bars are shown in the lower right corner in (A). Data are presented as mean ± SD, n = 3. **P < 0.01; ns, not significant. (S2_Fig.TIF) [file ppat.1013903.s002.tif]

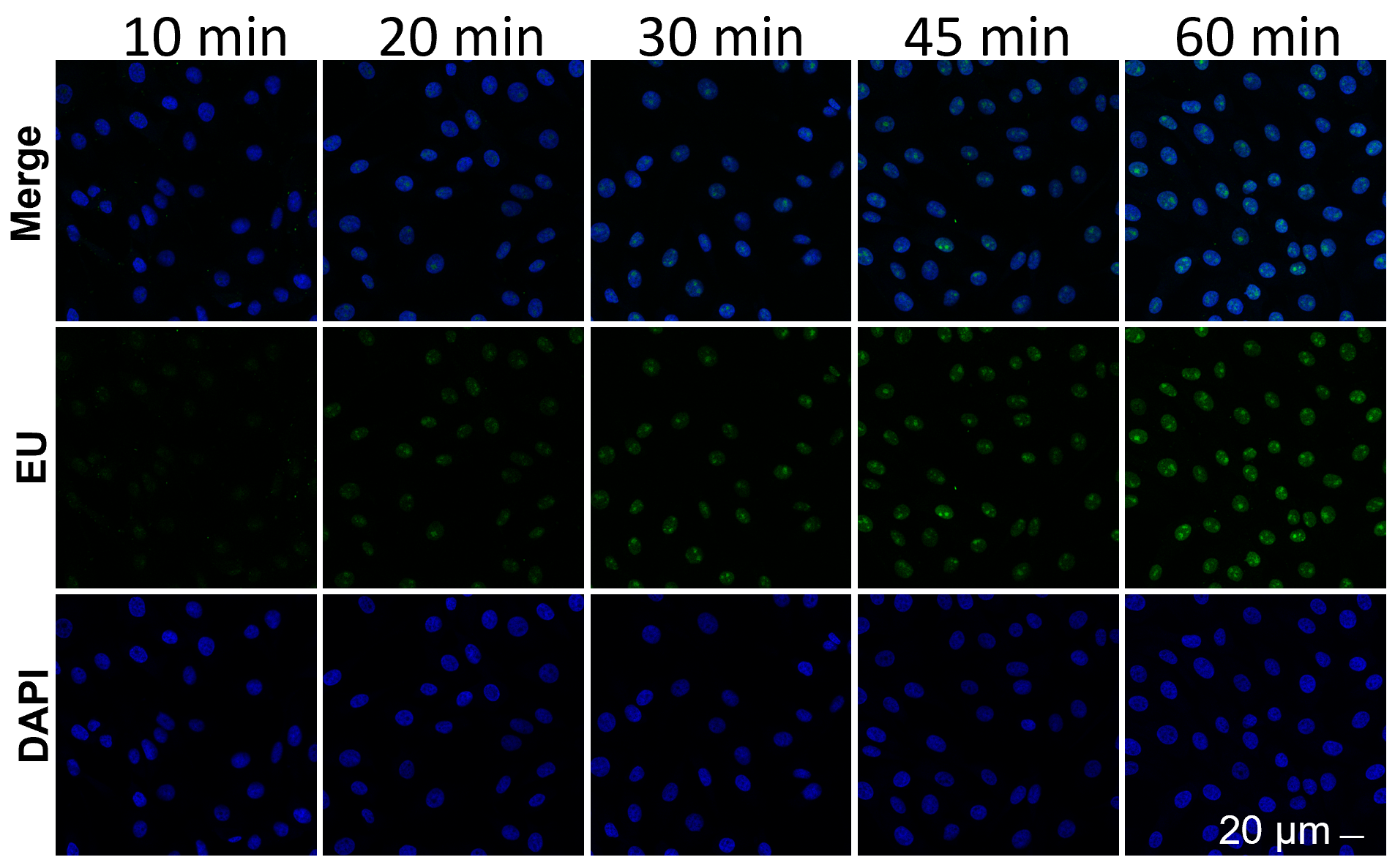

Supplement: S3 Fig — EU was added at a final concentration of 100 μM to MDBK cells, and the distribution of RNA in the cells was detected by laser confocal microscopy at 10 min, 20 min, 30 min, 45 min and 60 min after addition, respectively. The data are representative of results from three independent experiments. (S3_Fig.TIF) [file ppat.1013903.s003.tif]

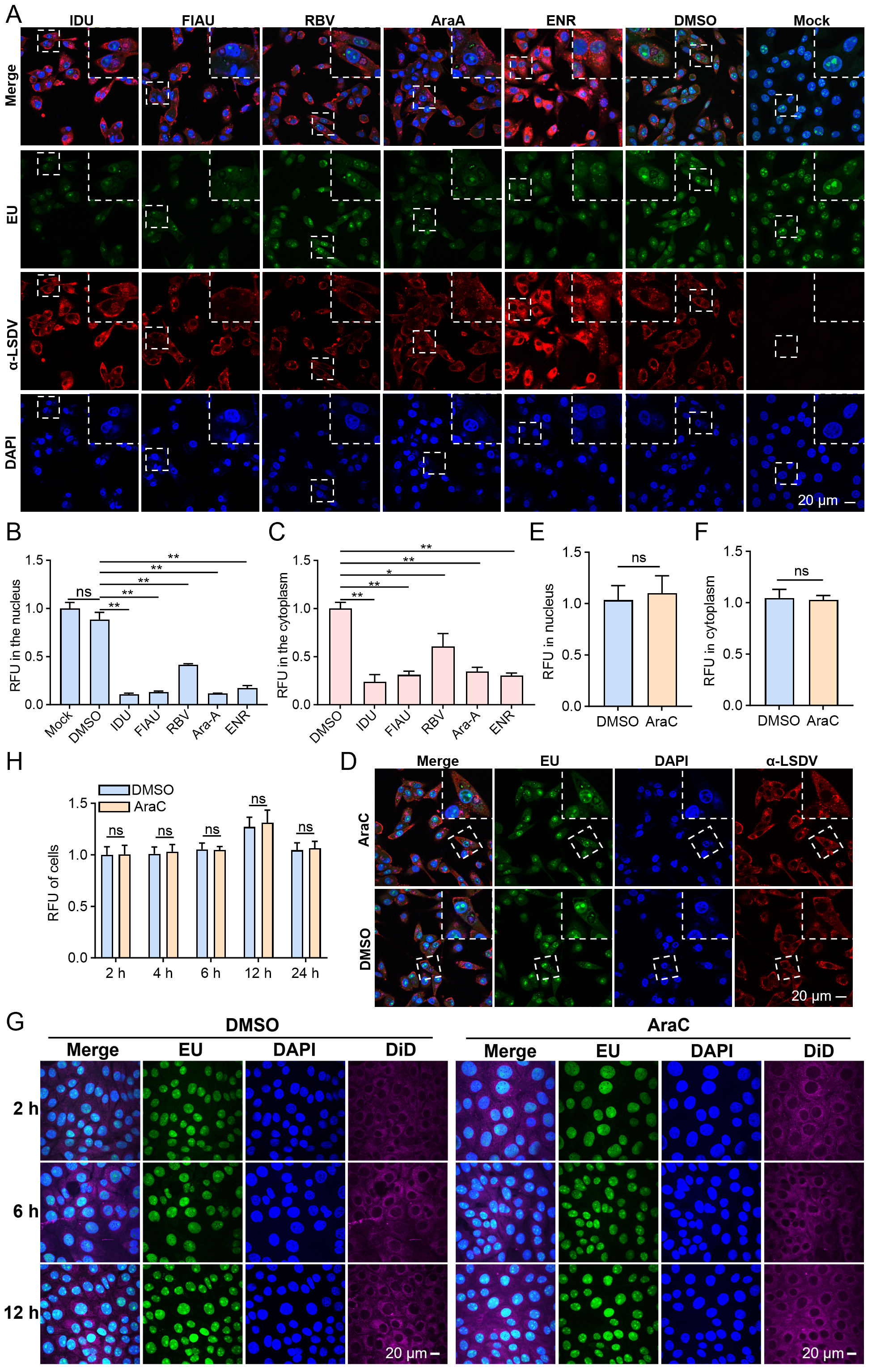

Supplement: S4 Fig — (A-C) MDBK cells were infected with LSDV at 1 MOI. At 24 hpi, cells were treated with ENR (100 μM), IDU (20 μM), FIAU (5 μM), RBV (20 μM), or AraA (20 μM) for 1 h, followed by an additional 1 h incubation with the same compounds in the continued presence of EU to label newly synthesized RNA (A). Viral proteins were detected using rabbit anti-LSDV polyclonal antibodies. Nuclear (B) and cytoplasmic (C) RFU values were quantified using ImageJ. (D-F) MDBK cells were infected with LSDV at 1 MOI. At 24 hpi, cells were treated with AraC (0.5 μM) for 1 h, followed by an additional 1 h incubation with AraC (0.5 μM) in the continued presence of EU to label newly synthesized RNA (D). Viral proteins were detected using rabbit anti-LSDV polyclonal antibodies. Nuclear (E) and cytoplasmic (F) RFU values were quantified using ImageJ. (G and H) MDBK cells were treated with AraC (0.5 μM) for 1 h, followed by incubation for the indicated times with AraC (0.5 μM) in the continued presence of EU to label newly synthesized RNA (G). The cytoplasm and plasma membrane were stained with DiD. RFU values were quantified using ImageJ. Scale bars are shown in the lower right corner in (A, D and G). Data are presented as mean ± SD, n = 3. *P < 0.05; **P < 0.01; ns, not significant. (S4_Fig.TIF) [file ppat.1013903.s004.tif]

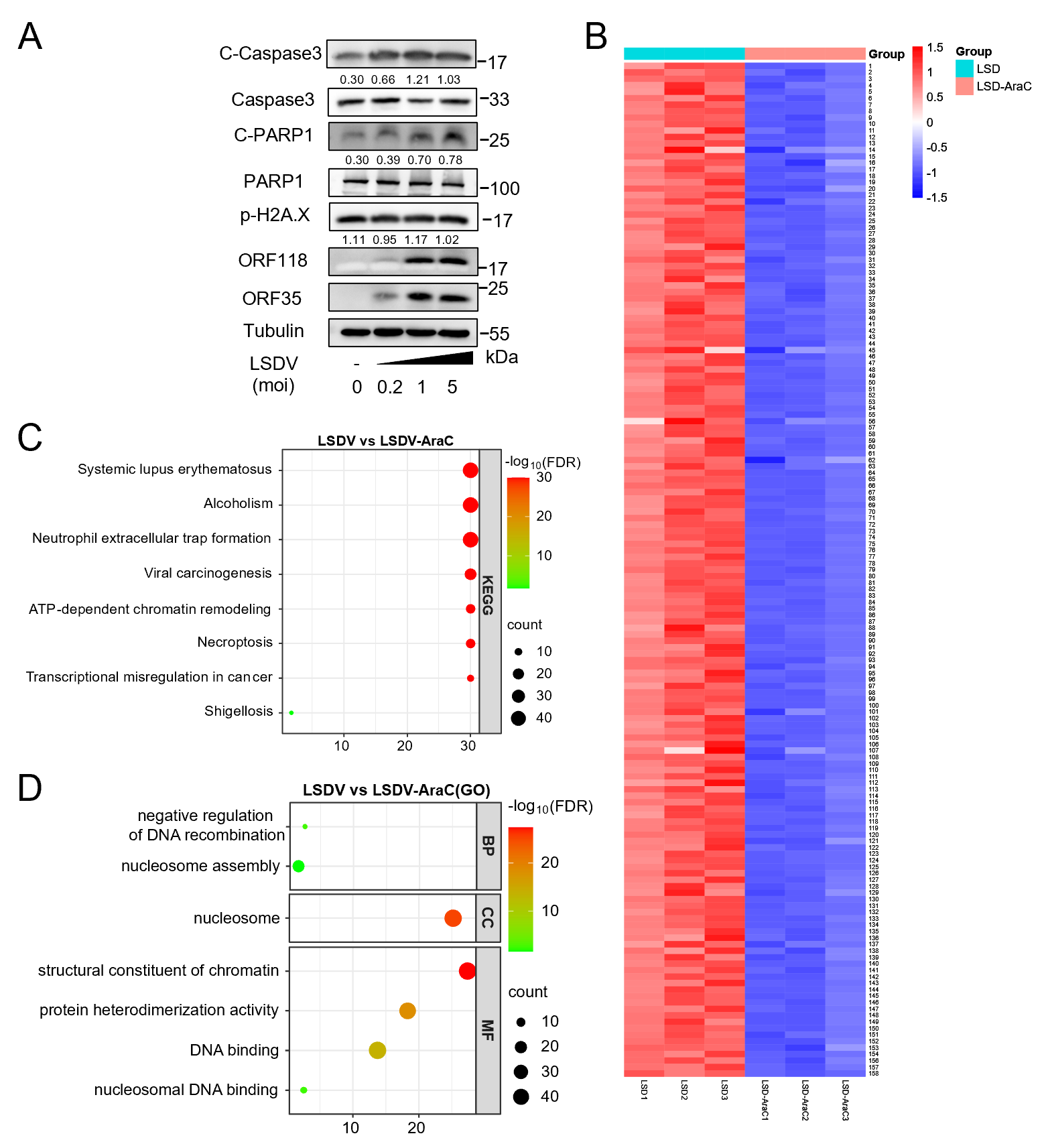

Supplement: S5 Fig — (A) MDBK cells were mock-infected or infected with LSDV at an MOI of 0.2,1 and 5 for 48 h; cell lysates were harvested for western blotting with the indicated antibodies. (B) Heatmap showing the fold-change differences of all viral ORF genes from LSD vs LSD-AraC in the RNA-seq experiment. (C and D) KEGG pathway and GO enrichment of DEGs from LSD vs LSD-AraC. (S5_Fig.TIF) [file ppat.1013903.s005.tif]
